# Single-cell triple omics sequencing reveals genetic, epigenetic, and transcriptomic heterogeneity in hepatocellular carcinomas

**DOI:** 10.1038/cr.2016.23

**Published:** 2016-02-23

**Authors:** Yu Hou, Huahu Guo, Chen Cao, Xianlong Li, Boqiang Hu, Ping Zhu, Xinglong Wu, Lu Wen, Fuchou Tang, Yanyi Huang, Jirun Peng

**Affiliations:** 1 Biodynamic Optical Imaging Center, College of Life Sciences, Peking University, Beijing 100871, China; 2 Department of Surgery, Beijing Shijitan Hospital, Capital Medical University, Beijing 100038, China; 3 Ninth School of Clinical Medicine, Peking University, Beijing 100038, China; 4 School of Oncology, Capital Medical University, Beijing 100038, China; 5 Ministry of Education Key Laboratory of Cell Proliferation and Differentiation, Peking University, Beijing 100871, China; 6 Peking-Tsinghua Center for Life Science, Beijing 100084, China; 7 Center for Molecular and Translational Medicine (CMTM), Beijing 100101, China; 8 College of Engineering, Peking University, Beijing 100871, China

**Keywords:** scTrio-seq, CNV, transcriptome, DNA methylome, HCC

## Abstract

Single-cell genome, DNA methylome, and transcriptome sequencing methods have been separately developed. However, to accurately analyze the mechanism by which transcriptome, genome and DNA methylome regulate each other, these omic methods need to be performed in the same single cell. Here we demonstrate a single-cell triple omics sequencing technique, scTrio-seq, that can be used to simultaneously analyze the genomic copy-number variations (CNVs), DNA methylome, and transcriptome of an individual mammalian cell. We show that large-scale CNVs cause proportional changes in RNA expression of genes within the gained or lost genomic regions, whereas these CNVs generally do not affect DNA methylation in these regions. Furthermore, we applied scTrio-seq to 25 single cancer cells derived from a human hepatocellular carcinoma tissue sample. We identified two subpopulations within these cells based on CNVs, DNA methylome, or transcriptome of individual cells. Our work offers a new avenue of dissecting the complex contribution of genomic and epigenomic heterogeneities to the transcriptomic heterogeneity within a population of cells.

## Introduction

The development of single-cell genome, DNA methylome, and transcriptome sequencing technologies in recent years has greatly aided dissection of the heterogeneity within a population of cells^1,2^. We and others have developed single-cell RNA-seq methods, such as scRNA-seq, Smart-seq/Smart-seq2, CEL-seq, MARS-seq, STRT-seq, and Quartz-seq^3,4,5,6,7,8^, and applied these techniques to analyze gene expression dynamics during mammalian embryonic development or tumor heterogeneity^9,10,11,12,13^. Single-cell genome-sequencing technologies have been used to reveal recombination patterns and aneuploidies in single human germ cells^14,15^, and genomic heterogeneities in tumors and circulating tumor cells^16,17,18^. Recently, we and others have developed single-cell DNA methylome sequencing techniques, such as single-cell reduced representation bisulfite sequencing (scRRBS) and single-cell bisulfite sequencing (scBS)^19,20^. We have applied scRRBS in analyzing DNA methylome dynamics during mammalian early embryonic development^21^. Combined genome and transcriptome analyses of a single cell based on either microarray or next-generation sequencing have also been successfully used to analyze tumor heterogeneity^22,23,24,25^. However, to directly analyze the mechanisms by which genetic and epigenetic factors regulate gene expression in an individual cell, the genome, epigenome, and transcriptome need to be simultaneously analyzed in a single cell. This approach is especially desirable for cancer, which displays strong heterogeneity in all of these three omics^26,27,28^.

Here we report the development of a single-cell triple omics sequencing technique, single-cell triple omics sequencing (scTrio-seq), and the application of this technique for analyzing the relationship between the genome (copy-number variations, CNVs), DNA methylome, and transcriptome of a single mammalian cell. We demonstrate that CNVs can be reliably identified using single-cell RRBS data produced from the scTrio-seq assay. We observed a negative correlation between promoter methylation and RNA expression, and a positive correlation between gene body methylation and RNA expression, in a single cell. Furthermore, a strong positive correlation between the DNA copy number and gene expression within the affected genomic region was found. In contrast, the DNA copy number does not affect DNA methylation level of the region. Finally, we used scTrio-seq to analyze 25 single cells derived from a human hepatocellular carcinoma (HCC) tissue sample and found two subpopulations distinct in DNA copy numbers, DNA methylation, or RNA expression levels. By comparing the multi-omic differences between two HCC subpopulations, we found that the subpopulation I, accounting for a minor part in tumor tissues, harbored more copy-gain CNVs, expressed more invasive cell markers, and were more likely to evade immune surveillance.

## Results

### Development of the scTrio-seq method

**Figure 1:**
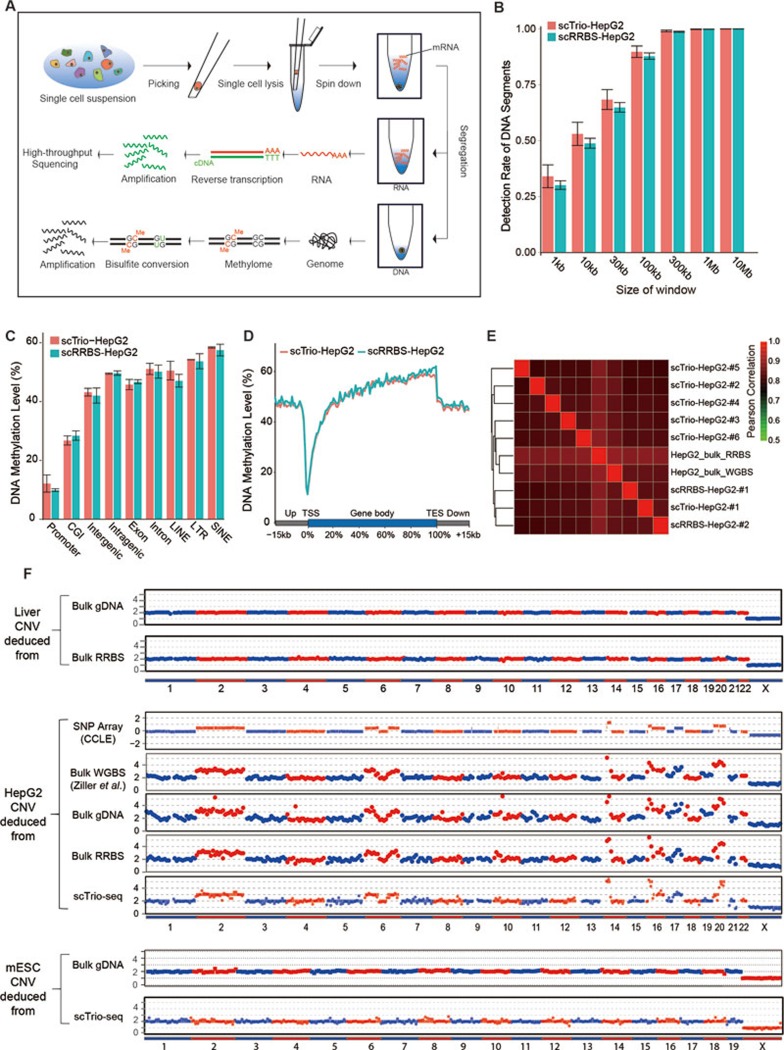
Sensitivity and reliability of the scTrio-seq technique. **(A)** A flow chart illustrating the scTrio-seq technique. After a single cell was lysed with mild lysis buffer, the lysis product was centrifuged. The supernatant was transferred to a new tube for transcriptome sequencing analyses, while the pellet (containing the nucleus) was bisulfite-converted for genome (CNVs) and epigenome sequencing analyses. **(B)** Comparing the rate of detection of DNA segments in HepG2 scTrio-seq data and HepG2 scRRBS data. The total DNA segments are those that can be detected in the bulk HepG2 RRBS data. **(C)** Comparing the average DNA methylation levels of CpG sites in different genomic regions between HepG2 scTrio-seq and HepG2 scRRBS data. **(D)** DNA methylation pattern in gene body regions as determined from HepG2 scTrio-seq data and RRBS data. The averaged DNA methylation level of CpG sites is calculated from all RefSeq genes in regions from the TSSs to TESs and their 15-kb flanking regions. **(E)** Unsupervised hierarchical clustering analysis based on Pearson correlations between global CpG methylation levels of different HepG2 samples. **(F)** CNV deduction results at a 10-Mb resolution. The normalized copy number values (red or blue dots) for the bulk genome DNA sequencing data and bulk RRBS data are shown. For the scTrio-seq data, HMM fitting results (red or blue segments) are also shown.

First, we developed a mild lysis protocol with which we only lysed the cytoplasm of an individual cell to release the mRNAs into the solution while keeping the nucleus intact. We next centrifuged the lysis product to separate the mRNA-containing supernatant from the nucleus-containing precipitate, and each was transferred to a different tube. The supernatant was subjected to the scRNA-seq method we previously developed^29^, whereas the precipitate was subjected to DNA methylome sequencing using the scRRBS method we recently developed^19^. This approach simultaneously yielded genomic (in term of CNVs), DNA methylomic, and transcriptomic information from the same cell. We have named this new method the scTrio-seq technique (Figure 1A).

To test the method, we sequenced six single HepG2 cells (a human hepatoblastoma-derived cell line) and six mouse embryonic stem cells (mESCs) using scTrio-seq; we also subjected HepG2 cells and mESCs to scRNA-seq and scRRBS as technique controls. The DNA methylome data obtained from scTrio-seq yielded an average of 1.5 million CpG sites from a single HepG2 cell and 0.8 million CpG sites from a single mESC cell, which is comparable to the detection efficiency of the standard scRRBS method (Table 1 and Supplementary information, Table S1). Compared with scRRBS data, the scTrio-seq technique did not result in a significant loss of DNA segments, even at a resolution of 1 kb (Figure 1B). This result demonstrates that the physical separation of the nucleus from the supernatant can retain all the chromosomes of an individual cell. Moreover, the DNA methylation levels of individual HepG2 cells analyzed by scTrio-seq and those analyzed using standard scRRBS technique were also comparable (Figure 1C and Supplementary information, Figure S1A). We were able to detect the hypomethylation valleys around transcription start sites (TSSs) as well as hypermethylation patterns of the gene bodies at the single cell level (Figure 1D and 1E, Supplementary information, Figure S1B). These results indicate that the measurement of the DNA methylome by scTrio-seq is as accurate as that obtained by the standard scRRBS approach.

We next tested the ability of scTrio-seq to accurately measure the gene expression pattern. We found that scTrio-seq detected an average of 6 179 genes in a single HepG2 cell. When we merged the transcriptome sequencing data from only six individual cells analyzed by scTrio-seq, a total of 10 390 genes were detected; this detection efficiency is comparable to that of a standard RNA-seq for bulk cells (Supplementary information, Figure S1C and S1D). The correlation between the scTrio-seq data and the standard scRNA-seq data was quite high (Pearson correlation coefficient = 0.96; Supplementary information, Figure S1E). To confirm the accuracy of quantitative gene detection using scTrio-seq, we quantified the relative gene expression using real-time quantitative PCR (qPCR) as previously described^30^. The results showed that scTrio-seq quantified the expression of genes highly accurately when only half of the single-cell lysis product was used for cDNA amplification (Supplementary information, Figure S1F).

### CNV deduction using scTrio-seq

Next, we attempted to identify global CNV patterns using the standard RRBS data of bulk cells, which could cover 0.57 million unique DNA fragments after MspI digestion at CCGG......CCGG. This corresponds to ∼1 900 unique MspI-digested DNA fragments per 10-Mb bin that can support the copy-number measurement. We observed strong correlations between the sequencing depth and GC content, especially the number of MspI-digested DNA fragments in each 10-Mb bin (Supplementary information, Figure S2A). We also performed both RRBS and whole-genome sequencing of the normal human liver tissues as a normal control. The same CNV patterns were observed in both the HepG2 whole-genome sequencing data and in the bulk RRBS data at a resolution of 10 Mb, consistent with that of the published SNP array data (Cancer Cell Line Encyclopedia)^31^ and whole-genome bisulfite sequencing data^32^ (accession number SRX332734; Figure 1F). We also calculated the sensitivity and specificity at different resolutions using the whole-genome sequencing data as a standard reference (Supplementary information, Figure S2B and S2C).

As a single mammalian cell has only two copies of genomic DNA, it is not feasible to simultaneously perform single-cell whole-genome DNA amplification and bisulfite conversion of the genome. Because more than one hundred thousand MspI-digested DNA fragments were recovered in the DNA methylome data of scTrio-seq (Table 1), we tested the ability to measure CNVs using the RRBS data of scTrio-seq in an individual cell. We also observed strong correlations between the sequencing depth and the number of MspI-digested DNA fragments for each 10-Mb bin in the scTrio-seq and scRRBS data (Supplementary information, Figure S3A and S3B). After normalization by the normal liver tissue, scTrio-seq can also be used to accurately deduce almost all the CNVs in a single HepG2 cell at a 10-Mb resolution (Figure 1F).

We verified the diploid nature of our mESC cell line by normalizing it with the published normal diploid mESC data (accession number SRX673789, Figure 1F and Supplementary information, Figure S3C). Using the bulk mESC cell data as a control, we observed a 95.3% specificity and 98% sensitivity of CNV deductions at a 10-Mb resolution using scTrio-seq data (Supplementary information, Figure S3D). Furthermore, we also fitted the normalized copy numbers to integer values for scTrio-seq data and scRRBS data using a hidden Markov model (HMM)^16,33^. As the RRBS reads are not uniformly distributed in the genome, we tried to raise the resolution of CNV deduction of scTrio-seq by focusing on the highly covered genomic regions, and found that shorter CNV segments and more accurate breakpoints of CNVs can be identified (Supplementary information, Figure S3E). Together, these results demonstrate that the scTrio-seq technique can simultaneously and accurately analyze the genome (CNVs), DNA methylome, and transcriptome in a single cell.

### Application of scTrio-seq to explore the relationship between the genome (CNVs), DNA methylome, and transcriptome in a single cell

**Figure 2:**
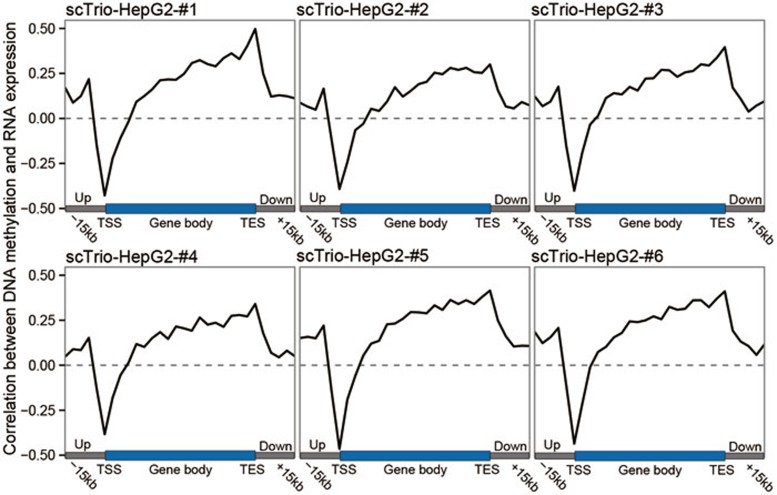
The relationships between DNA methylation and gene expression in single cells. The Pearson correlations between DNA methylation and gene expression are calculated in different regions on gene body (from TSS to TES) and their 15-kb flanking regions in scTrio-seq data of HepG2 cells.

Epigenetic modifications are important for regulating chromatin status and gene expression. They are potentially heterogeneous within a cell population, especially in cancer^34^. Many studies have indicated that DNA methylation in the promoter regions often negatively correlates with gene expression, whereas DNA methylation in the gene body positively correlates with gene expressions^35^. We also observed same correlations in bulk cell data (Supplementary information, Figure S4A). We next used scTrio-seq data to explore the relationship between methylation and gene expression in individual cells, and found a similar negative correlation between promoter DNA methylation and the expression level of the corresponding gene in each HepG2 cell (Figure 2 and Supplementary information, Figure S4B). This correlation indicates that high DNA methylation in promoters may repress the expression of corresponding genes within a HepG2 cell. Moreover, DNA methylation in the gene body (excluding promoter regions) showed a positive correlation with gene expression (Figure 2). Furthermore, this positive correlation increased when moving to the 3′-end of gene body, indicating that gene body DNA methylation may promote the transcription of these genes. To our knowledge, this is the first global demonstration of relationship between DNA methylation and RNA expression in single cells.

**Figure 3:**
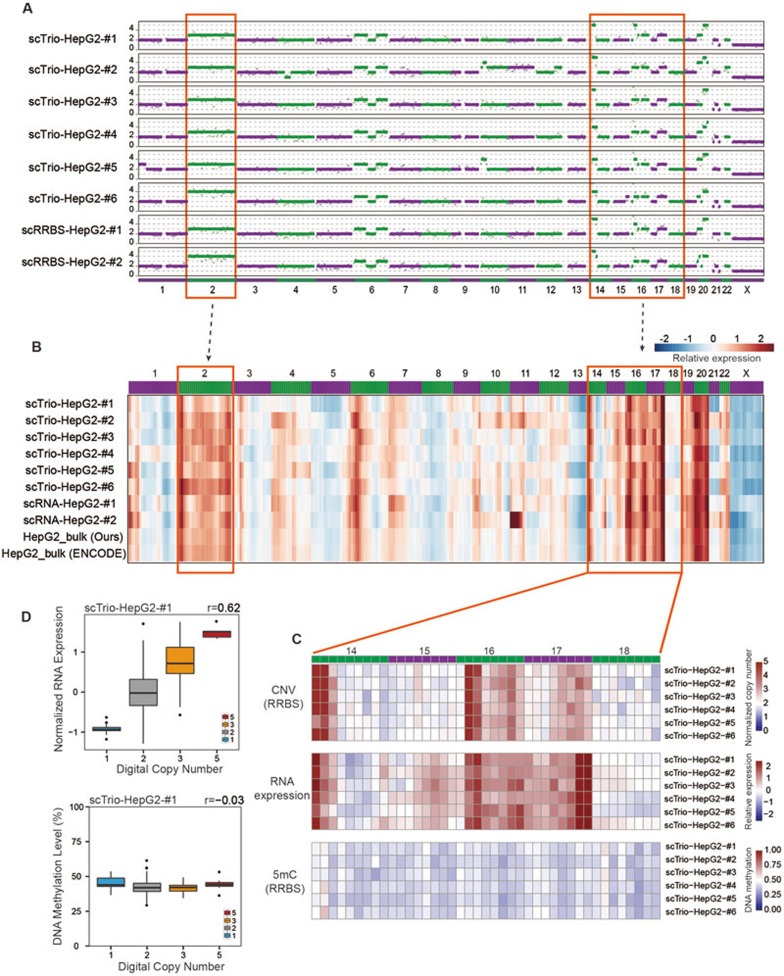
The relationships between genome (CNVs), DNA methylation, and gene expression in single cells. **(A)** CNV deductions of single HepG2 Trio-seq data or scRRBS data at a 10-Mb resolution. The purple or green dots represent the normalized copy numbers and the purple or green segments represent the integer copy number fitted by HMM. **(B)** Heat map of normalized relative gene expression levels in 10-Mb genomic windows. The genes are ranked according to their genomic positions. The relative expression values (normalized to liver bulk RNA-seq data) of all the genes in each 10-Mb window of each sample are represented by blue to red colors. **(C)** Relationship between CNV patterns, DNA methylation, and gene expression within single cells. Normalized DNA methylation value is compared with bulk liver RRBS data. Zoom-in pictures represent the normalized copy number, relative RNA expression, and DNA methylation values of the same 10-Mb window from the regions from Chr.14 to Chr.18. **(D)** The correlations between the copy numbers and gene expression (or DNA methylation levels). The boxplot shows the distributions of each 10-Mb window's relative expression level (or DNA methylation level) within each copy-number group. The Pearson correlation coefficient is shown at the top right corner. Note that there is no 10-Mb DNA fragments with digital copy number of 4.

The CNV patterns in the scTrio-seq data from six HepG2 cells showed that these six cells shared most of their CNVs. However, some CNVs were unique to only one of the samples (Figure 3A). For example, four copies of chromosome 2 were identified in sample scTrio-HepG2-#6, but only three copies of this region were identified in each of the other five single cells. Furthermore, we calculated the relative expression levels within each 10-Mb window of scTrio-seq data by normalizing the data with the RNA-seq data from normal liver tissues (Figure 3B). We compared the CNV patterns with the RNA expression patterns and found that expression of genes within the genomic regions with extra copies also increased proportionally. Similarly, the expression of genes within genomic regions with lost copies proportionally decreased (Figure 3A-3C). We observed a Pearson correlation coefficient of 0.68 ± 0.07 (mean ± SD) between the digital DNA copy number values and the gene expression levels in the same cell at a 10-Mb resolution, which is consistent with the bulk cell data (Figure 3D and Supplementary information, Figures S4C and S5A). These results indicate that CNVs contribute to the changes of gene expression by changing the copy numbers and dosages of genes within these genomic regions.

In contrast, the DNA methylation level within the genomic region that gained or lost copies showed no alteration (Figure 3C). The correlation coefficient between the digital DNA copy number values and DNA methylation levels was 0.05 ± 0.02 (mean ± SD) for single cells (Figure 3D and Supplementary information, Figure S5B). For the bulk HepG2 data, there was no correlations between DNA copy numbers and DNA methylation levels even at a 0.5-Mb resolution (Supplementary information, Figure S4C), indicating that large-scale CNV patterns generally did not directly affect the DNA methylation levels within the corresponding genomic regions. Therefore, at the single-cell resolution, CNVs affect gene expression mainly by dosage effect but do not markedly change the DNA methylation patterns of the corresponding genes.

### Application of scTrio-seq to explore the genome (CNVs), DNA methylome, and transcriptome relationships in HCC

Single-cell analyses have provided new insights into the evolution, therapeutic responses, and drug resistance of cancer^16,36^. Single-cell genome and transcriptome sequencing analyses have accelerated studies of tumor heterogeneity^37^. Changes in DNA methylation also have a critical role in tumorigenesis^38,39^. Global DNA hypermethylation or hypomethylation has been observed in many types of cancers, and drugs that regulate DNA methylation, such as 5-azacitidine and decitabine, have been used in cancer therapy^40,41^. However, the heterogeneity of DNA methylation in tumors *in vivo* has not been well characterized over the entire genome at single-cell resolution, and the relationships between the genome, epigenome, and transcriptome in single cancer cells have not been directly elucidated.

**Figure 4:**
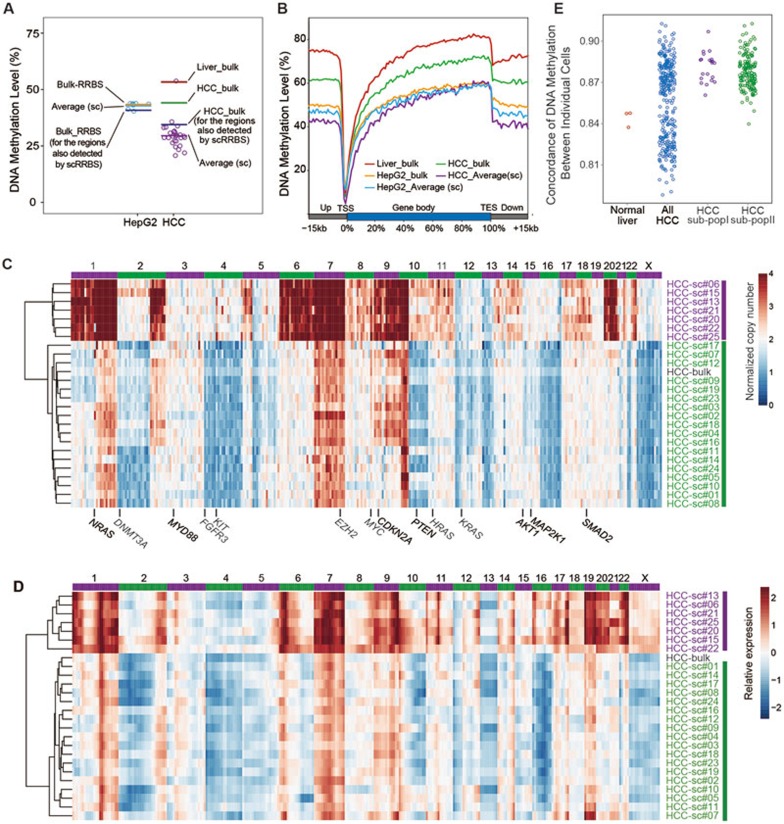
ScTrio-seq analyses of single HCC cells. **(A)** Global DNA methylation levels of CpG sites of HepG2 cells and HCC cells. Each circle represents the DNA methylation of one single cell, and the lines represent the bulk or average (single-cell) results. HCC bulk (for the regions also detected in scRRBS) represents the DNA methylation of HCC-bulk cells, the calculation for which only includes regions that are also detected in the HCC scTrio-seq data. **(B)** Average CpG methylation levels in gene bodies (from TSSs to TESs) of all RefSeq genes and their 15-kb flanking regions in HepG2 cells and HCC cells. **(C)** Heat map showing normalized copy-number values of 10-Mb windows deduced from RRBS data of scTrio-seq analysis. The HCC cells are clustered based on their CNV patterns. **(D)** Heat map showing relative gene expression levels in each 10-Mb genomic window. The HCC cells are clustered based on their expression levels in each genomic window. **(E)** The concordance of the DNA methylation of normal liver cells and that of HCC cells. Each dot shows the Pearson correlation coefficient between any two single cells within each group.

The RRBS data obtained from bulk HCC cells indicated global hypomethylation compared with the adjacent normal liver cells (Supplementary information, Figure S6), which is consistent with results from previous studies^42,43^. We next analyzed 26 single cells isolated from a HCC sample from one patient using the scTrio-seq technique. As expected, these HCC cells showed global hypomethylation patterns (Figure 4A and 4B), except for one cell (HCC-sc#26; Supplementary information, Figure S7). Unlike the other 25 cells, this cell lacked significant aneuploidies (Supplementary information, Figure S8), indicating that it was likely to be noncancerous cell. After excluding this cell, we focused on the remaining 25 cancer cells in further analyses.

As observed in HepG2 cells, the DNA copy number and expression profile also showed strong correlations in HCC cells, with a Pearson correlation coefficient of 0.73 ± 0.04 (mean ± SD) between the digital copy-number values and the gene expression levels in individual HCC cells. However, the DNA copy number did not significantly affect the DNA methylation at the 10-Mb scale (Pearson correlations, 0.025 ± 0.035; Supplementary information, Figure S7C).

### Differences in triple-omics between two subpopulations of HCC cells

We then performed an unsupervised hierarchical clustering analysis of these 25 hepatocellular carcinoma cells based on their CNV patterns, and this separated these cells into two subpopulations. All the 25 HCC cells harbored extra copies of Chr. 7 and the q arm of Chr. 1; these extra copies were also detected in several previously analyzed HCC samples^44^. Furthermore, subpopulation I harbored several unique CNVs including gained copies of Chr. 8, Chr. 11 and Chr. 20. Conversely, subpopulation II lost copies of Chr. 4 and Chr.16 (Figure 4C and Supplementary information, Figures S8 and S9A). We also identified similar patterns and obtained similar clustering results using RNA expression values of the genes within each 10-Mb window (Figure 4D). At a 10-Mb resolution, a comparison of the CNV patterns between subpopulation I and subpopulation II defined 164 10-Mb bins with CNV differences between the two subpopulations as “differential CNV regions” and 158 bins without CNV differences as “shared CNV regions”.

**Figure 5:**
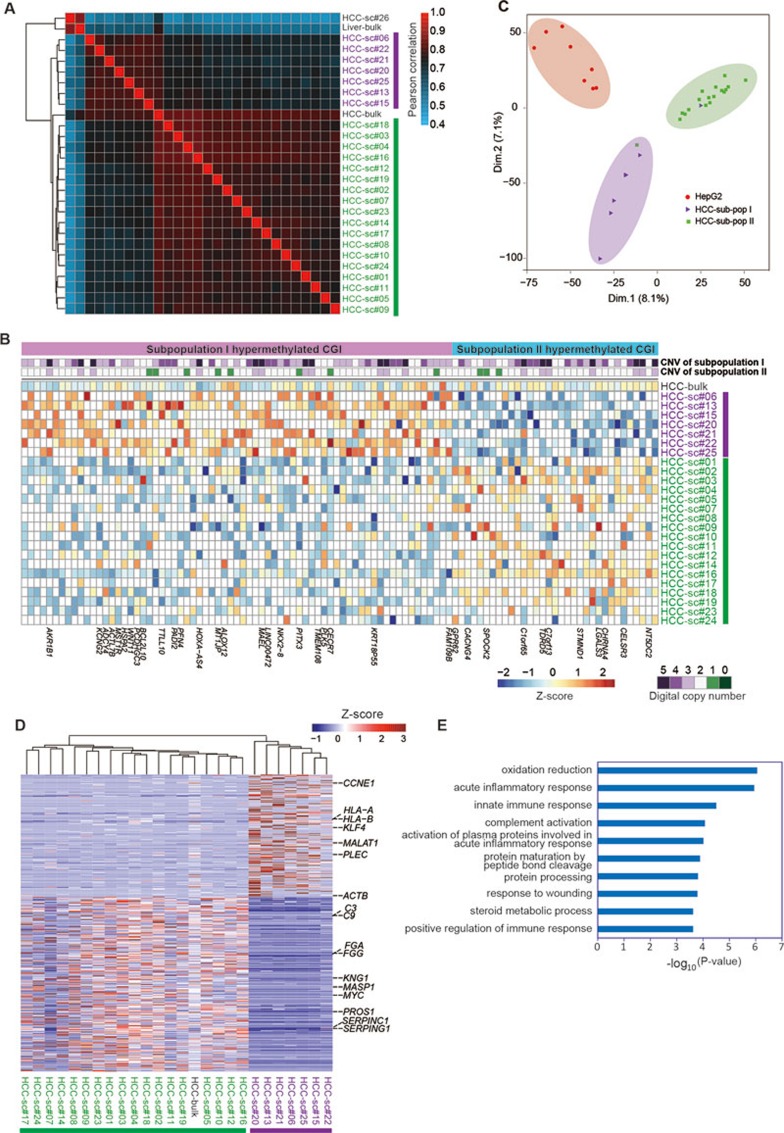
Differences in triple omics between subpopulation I and II of HCC cells. **(A)** Unsupervised hierarchical clustering analysis based on the Pearson correlations of CpG methylation levels between different HCC samples. A “pairwise” method is used when calculating the Pearson correlations. **(B)** Differentially methylated CGIs (dmCGIs) between subpopulation I and subpopulation II HCC cells. The DNA methylation level of each CGI is normalized using the Z-score. The white squares represent the CGIs that are not detected in each sample. The genes with a dmCGI within the ±1 kb regions of their TSS regions are labeled at the bottom. **(C)** Principal component analysis of the HepG2 and HCC cells according to the expression level of RefSeq gene. **(D)** Hierarchical clustering of Pearson correlation between single HCC single cells considering the genes that are differently expressed between subpopulation I and subpopulation II. The expression level of each gene is normalized using the Z-score. The genes in the complement and coagulation cascades and several other cancer-related genes are marked. **(E)** Gene Ontology (GO) analyses of the genes whose expressions are downregulated in subpopulation I.

Next, we examined the heterogeneity in global DNA methylation level among these 25 cancer cells. We found that cells of the same subpopulation (I or II) had higher correlations compared with the normal liver cells, while there was noticeable heterogeneity among two subpopulations (Figure 4E). We then performed unsupervised clustering analysis for these cells based on methylation level of all detected CpG sites. Notably, this analysis also separated these cells into two subpopulations exactly identical to those identified by the CNV patterns (Figure 5A and Supplementary information, Figure S9B). Of these two subpopulations, subpopulation I displayed slightly higher level of global DNA methylation than subpopulation II, but the differentially methylated regions (DMRs) were not associated with the regions of different CNV (Supplementary information, Figure S9C and S9D).

To analyze the DMRs between and within two HCC subpopulations, we calculated the methylation level and variance with a 3-kb sliding window across all the 25 HCC cells^20^. After ranking the windows with their DNA methylation variances among the 25 HCC cells, we found the top variable windows were significantly enriched in the CpG island (CGI) region (Fisher's exact test, FDR = 2 × 10^−15^; Supplementary information, Figure S10A). Moreover, the CGI region also had higher DNA methylation variances within each subpopulation. A total of 140 and 200 out of the 300 most variable windows were located in CGI regions in subpopulation I and subpopulation II, respectively (Supplementary information, Figure S10B and S10C). We next compared the DNA methylation level of each CGI and identified the CGIs with significant methylation level difference (difference > 0.3; Fisher's exact test *P* value < 0.05) as differentially methylated CGIs (dmCGIs) between the two HCC subpopulations. We found 69 CGIs were hypermethylated in subpopulation I, and 33 were hypermethylated in subpopulation II (Figure 5B).

To analyze gene expression differences between the two subpopulations in HCC, we performed a principal component analysis using the gene expression data from 25 HCC cells and this again notably distinguished two cell subpopulations consistent with the results from the analyses on CNV patterns and DNA methylation data (Figure 5C). Subpopulation I expressed significantly higher levels of 245 genes and significantly lower levels of 350 genes than subpopulation II (FDR < 0.05; Figure 5D). The 245 genes with higher expression levels in subpopulation I were not significantly enriched in Gene Ontology (GO) terms. Interestingly, the 350 genes expressed significantly lower in subpopulation I were clearly enriched in several GO terms, such as acute inflammatory response, innate immune response, and complement activation, as well as complement and coagulation cascades in the KEGG pathway analysis (Figure 5E and Supplementary information, Figure S11). Complement activation has been considered a biomarker of many tumors^45^, and the protein AIM has been identified as the complement activator that initiates HCC necrotic death^46,47^. Thus, the data suggest that the cells in subpopulation I are less responsive to the immune recognition than those in subpopulation II.

**Figure 6:**
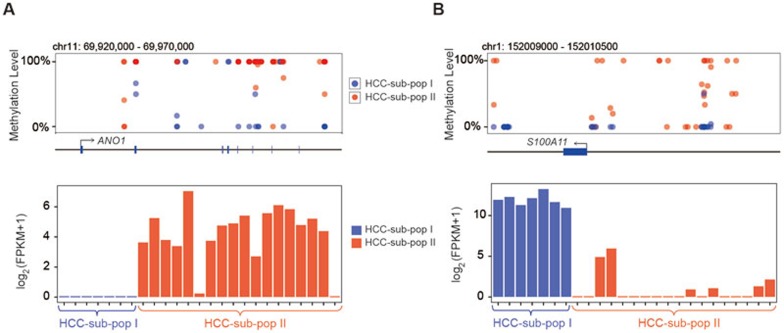
Differentially methylated regions on gene promoter and gene body regulate differentially expressed genes. **(A)** The DNA methylation levels on the gene body regions of *ANO1* (NM_018043) are much lower in subpopulation I HCC cells. Consequently, *ANO1* (NM_018043) has lower expression levels (log_2_ (FPKM + 1)) in subpopulation I cells. **(B)** The DNA methylation levels on the promoter regions of *S100A11* (NM_005620) are much lower in subpopulation I HCC cells. Consequently, *S100A11* (NM_005620) has higher expression levels (log_2_ (FPKM + 1)) in subpopulation I cells.

Although DNA copy-number difference between two HCC subpopulations may contribute to differential RNA expression on a large scale of genomic region, the expression of individual genes is still regulated by DNA methylation in DMRs in a context-dependent manner. For example, both *ANO1* and *S100A11* have been reported to have important roles in tumorigenesis and cancer metastasis^48,49,50,51,52^. We found that for HCC cells in subpopulation I, both *ANO1* and *S100A11* had lower DNA methylation levels. However, the hypomethylation occurred in the gene body of *ANO1*, whereas in *S100A11* it was the promoter that was hypomethylated, and the expression level of *ANO1* in these HCC cells is suppressed, whereas the expression of *S100A11* is elevated in subpopulation I (Figure 6A and 6B).

Taken together, these results indicate that the DNA copy number, DNA methylome, and transcriptome significantly differ between subpopulations I and II. The differential CNV regions, DMRs, and differentially expressed genes regulated each other. HCC cells in subpopulation I, which harbor more copy-gain CNVs, are likely to escape the immune recognition and are more invasive compared with the cells in subpopulation II. It also should be noted that these single cells account for a minor part in the tumor tissue, and thus their unique genomic, epigenomic, and transcriptomic characters will be concealed in bulk analysis.

## Discussion

Cancer development and metastasis involve various aspects of genomic alternations, including but not limited to changes of genomic DNA, epigenetic modifications, gene expression, and complex interplays between them. The intrinsically strong intratumoral heterogeneity makes it difficult to define an accurate regulatory relationship among genome, epigenome, and transcriptome using bulk cells. Well-established single-cell methods were typically optimized to examine only one aspect of regulatory hierarchy, hence losing the possibility to probe the inter-omics regulations at the single-cell level. Recently reported single-cell dual-omics sequencing methods (e.g., DR-seq and G&T-seq)^24,25^ can depict regulatory relationships between genome and transcriptome, but are incapable to provide epigenetic information (methylome especially), which is critical to RNA expression regulation.

In this study, we have developed a novel method called scTrio-seq that can, for the first time, simultaneously acquire genome (CNVs), DNA methylome, and transcriptome information of the single cells. We have shown that this method can accurately deduce CNV patterns at a 10-Mb resolution, obtain the methylation patterns of 1.5 million CpG sites, and detect the expression levels of 6 179 genes on average in a single mammalian cell. Correlations between genomic (CNVs), methylomic, and transcriptomic data have also been analyzed in the same individual cells for the first time. Changes in gene dosages due to CNVs proportionally affect the RNA expression levels of the corresponding regions, whereas they do not significantly affect the DNA methylation levels in these regions.

In scTrio-seq, we physically separate the DNA and RNA molecules before amplifications and primer binding, eliminating the possible genomic DNA cross-contamination in scRNA-seq. Moreover, our mild lysis condition and separation procedure are compatible with conventional single-cell methods. For example, the DNA in the lysate can be used for single-cell whole-genome amplification, scRRBS or scBS analysis, while the RNA can be processed using Smart-seq or CEL-seq pipelines in parallel. Admittedly, to avoid disturbing the nuclear DNA precipitate, some RNA-containing supernatant is left in the tube after separation, leading to a slight loss of RNA transcripts. However, we found that using half of the lysate did not compromise the whole-transcriptome analysis. Further improvement may be achieved by increasing compactness of DNA pellet to optimize the separation of DNA and RNA.

Using scTrio-seq, we can detect subpopulations of cancer cells according to the genome (CNVs) information, and infer malignancy and metastasis potentials of the subpopulations based on triple-omic information. Moreover, we are also able to explore the relationships between differential CNV regions, differentially expressed genes, and DMRs. After filtering out the differences between subpopulations, we can unveil the heterogeneity existing within each subpopulation. Our work paves the way for deciphering the heterogeneity and complexity of cell populations in development and cancer by simultaneously interrogating the genome, methylome, and transcriptome of their constituents at the single-cell level.

## Materials and Methods

### Cancer sample collection and single-cell isolation

This study was approved by the Ethics Committee of Beijing Shijitan Hospital, Capital Medical University. Surgically removed HCC specimens were collected from a 51-year-old male patient who had provided written informed consent. All the clinicopathologic results of specimen are accordant with hepatocellular carcinoma. The pathological report shows that the tumor has extensive degeneration and necrosis, the surrounding tissue of the tumor has nodular cirrhosis, and the pathological features are accordant with hepatitis B-associated cirrhosis. The IHC result of the specimen is AFP (±), GPC3 (−), ki-67 (−), and CD34 (+). The tissues were mechanically dissociated into small pieces on ice and then digested into an HCC cell suspension using a Tumor Dissociation Kit (Miltenyi Biotec 130-095-929); a part of the cancer samples was retained for bulk genome, transcriptome, and methylome sequencing. Three normal liver cells were obtained from the adjacent normal tissue of another HCC patient. The cell viability of digested HCC cells were tested with Propidium iodide (PI) staining. Among the digested HCC cells, 76.5% were PI negative (live cells) and CD45 negative (non-leukocyte cells) analyzed by FACS. The HepG2 cells were cultured in RPMI 1640 medium (Corning, 10-040-CVR) containing 10% fetal bovine serum under 5% CO_2_ at 37 °C. Before the single-cell study, HepG2 cells were digested with 0.5% trypsin into a single-cell suspension and picked with a mouth pipette.

### Purification of HCC cells by MACS

The digested HCC cell suspension was passed through a 70-μm strainer (BD Biosciences) and then passed through a 40-μm strainer (BD Biosciences) to obtain a single-cell suspension. The HCC cells were purified by magnetic-activated cell sorting (MACS) using MS columns in MACS buffer (2 mM EDTA, 0.5% BSA in PBS). Red blood cells and inflammatory infiltrate cells were depleted using CD45 and CD71 MACS beads (Miltenyi Biotec) and MS columns (Mitenyi Biotec).

### Single-cell segregation for DNA methylome and transcriptome sequencing

Single cells were individually transferred into 200-μl tubes using a mouth pipette. The single cells were lysed in 7 μl of soft buffer (500 mM KCl, 100 mM Tris-HCl (pH = 8.3), 1.35 mM MgCl_2_, 4.5 mM DTT, 0.45% Nonidet P-40 (Roche, 11332473001), 0.18 U SUPERase-In (Applied Biosystems, AM2694), and 0.36 U RNase-inhibitor (Applied Biosystems, AM2682) for 30 min at 4 °C, and then the lysate was vortexed for 1 min at room temperature. All RNAs were released, whereas the nucleus remained intact. The lysed single cell was then centrifuged at 1 000× *g* for 5 min to leave the nucleus at the bottom of the tube. The 4 μl of lysis product supernatant was carefully removed and added to another 200-μl tube containing spike-in RNA (ERCC, Ambion) and reverse transcriptase. This fraction was used for transcriptome analyses, whereas the remaining 3 μl of lysis solution (containing the nucleus) was used for genome (CNVs) and methylome analyses. The upper 4 μl of lysis solution was reverse-transcribed with poly T primers, and the cDNA was amplified as previously described^29^. Protease was added to the bottom 3 μl lysis solution, and the DNA was added with 60 fg of unmethylated lambda DNA (Fermentas). The released naked DNA was then digested and bisulfite-converted using the scRRBS method^19^.

### Sample quality control and library construction for sequencing

The cDNA amplicons of each single cell were quantified with qPCR of two housekeeping genes (*GAPDH* and *ACTB*). Amplified single-cell cDNA was purified with the DNA Clean & Concentrator 5 Kit (VisTech, HLLCTech, DC2005). The amplification primers were removed by selecting 500-3 000 bp cDNA products on a 2% agarose gel; the product was recovered from the gel using the VisClean Gel DNA Recovery kit (VisTech, HLLCTech, PC0313). The purified cDNAs were then sonicated with a Covaris S2 system to generate 150-250 bp fragments. The cDNA libraries were barcoded and amplified using NEBNext Ultra DNA Prep Kit for Illumina (New England Biolabs, E7370). Single-cell RRBS libraries were constructed according to previously published protocols^19^, and two genomic loci were checked in RRBS libraries with qPCR before the high-throughput sequencing to ensure that DNA was present. Only the libraries in which the two genomic loci were detected were sequenced. Bulk cDNA libraries and RRBS libraries were constructed according to previously published protocols^53^. All constructed libraries were used for 100-bp pair-end high-throughput sequencing on an Illumina HiSeq2000 or HiSeq 2500 Sequencer. The qPCR primers for checking the bisulfite-converted DNA were

Chr3_Forward: GTTAGGGAAGAGTTGGTTAGAG

Chr3_Reverse: TCTAAAACCAAATCTAAATCCTAAA

Chr17_Forward: GGTTTTTGGTGAGTTTTTTTT

Chr17_Reverse: AACCTACACAAACCCAAAAT

For the HepG2 and mESC cells, we picked 10 cells from each cell line. All 20 cells showed high RNA quality and DNA quality in qPCR quality control experiments. Then 6 out of the 10 cells of each cell line were sequenced using scTrio-seq technique, 2 cells were sequenced using scRNA-seq and 2 cells were sequenced using scRRBS. For the digested HCC cells, we picked 37 single cells, among which 9 cells showed low quality of cDNA and 2 cells showed low DNA quality in qPCR quality control experiments. We thus sequenced and analyzed the remaining 26 (70.3%) HCC single cells.

### Sequencing quality control and data processing

**Single-cell RNA seq data** The raw sequencing reads were trimmed to remove low-quality read ends, library construction adapters, and amplification primers. The clean reads were aligned to the human genome (hg19) or the mouse genome (mm9) with Tophat and the gene expression levels were calculated with Cufflinks^54^. Mapped reads, mapped ratio, and detected gene numbers are shown in the Supplementary information, Table S2. The number of detected RefSeq gene and NONCODE^55,56^ gene were calculated separately for each single-cell RNA-seq data, bulk cell data, as well as the published RNA-seq data of HepG2 cell line (ENCODE, ENCLB257SKY)^57^.

**Single-cell RRBS data** The raw sequencing reads were trimmed to remove low-quality read ends and library construction adapters. We then aligned the trimmed reads to human or mouse C-T (G-A) genomes with the Bismark software^58^. The bisulfite conversion rate for each sample, which is shown in the Supplementary information, Table S1, was calculated using a spike-in of unmethylated lambda DNA. The methylation level of each CpG site was then calculated by counting the methylated reads and unmethylated reads. Only the CpG sites with a depth of = 3 and a methylation level of ≥ 0.9 or ≤ 0.1 were used for further analyses of single-cell RRBS sequencing data.

### Calculation of the correlation between gene expression and DNA methylation at the single-cell level

The gene body was defined as the region from TSS to TES of each gene. Considering that the promoter regions and CGI regions in gene bod may influence the DNA methylation calculation of gene body, we excluded the promoter regions (from TSS to 2 000-bp downstream of TSS) and the CGI regions from each gene body region. The promoter region of each gene was defined as the region from 1 000-bp upstream to 500-bp downstream of the TSS. Only the genes with more than five CpG sites detected in the gene body region or gene promoter region were used to analyze the relationship with gene expression, and each CpG site used for analysis was required to be sequenced at depth of = 3. The DNA methylation level in the gene body or promoter region was calculated based on the mean methylation level of detected CpGs of each region. The gene expression level was the FPKM value calculated with Cufflinks program. The genes were then arranged according to their expression levels. The Pearson correlation coefficients (*r*) between gene body methylation or promoter methylation and the corresponding gene expression level (log_2_ (FPKM + 1)) were calculated as previously described^21^.

### Unique mappable MspI-digested fragments of RRBS data

We searched the reference genome (hg19 or mm9) for all possible MspI-digested fragments (CCGG......CCGG) except for the ones from random chromosomes. We then generated a simulated paired-end RRBS data using the sequences from two ends of these MspI-digested fragments. The simulated RRBS reads were mapped to the reference genome in the same manner as the experimental data were. We discarded the alignments that yielded multiple hits or that could have been mismatched by reads from elsewhere. After filtering, we defined 627 448 unique mapped fragments from 727 620 candidates in the human hg19 reference genome, and 339 101 unique fragments from 427 854 candidates in the mouse mm9 reference genome. Unique fragment counts in each genomic bin were calculated using BEDTools^59^.

### Correlations between DNA methylation and RNA expression

The gene body region (from TSS to TES) of each gene was divided into 20 equal fractions and the 15-kb upstream (or downstream) flanking regions were divided into five fractions. The mean DNA methylation level of CpG sites in each fraction of each gene was computed by Pearson correlation analysis with the corresponding genes. The genes with FPKM < 0.001 were reset to 0.001, and then the relative gene expressions (log_2_ (FPKM + 1)) were used for correlation analysis with DNA methylation levels of different genomic regions. The correlation of adult liver cells in Supplementary information, Figure S6C was calculated using the published whole-genome bisulfite sequencing data of adult liver cells (GSM916049) and the published RNA-seq data of human liver (accession number: ERX011229).

### CNV deduction with whole-genome sequencing data or RRBS data

Samtools depth was used to count the depth of each position across the genome. The total sequence depth of each window was counted, and then normalized using the total depth of each sample. The windows with low mappability such as centromere and telomere were not included in our analyzed windows. Because the systematic coverage bias in the RRBS data is too much to allow the deduction of copy numbers, we then normalized the sequence depth of each window by dividing it by the normalization factor. The normalization factor of each window was calculated by averaging the depth value of normal liver bulk RRBS data. The normalized copy number from each window was then used to cluster the human HCC cells with average-linkage hierarchical clustering.

To ensure that the mouse mESCs we used were normal diploid cells, we deduced the CNVs of bulk whole-genome sequencing data by normalizing it with the published normal diploid mESC data (accession number: SRX673789). For the single-cell RRBS data, the normalized copy numbers were further fitted to integer copy-number values using the hidden Markov model (HMM) as described for CNV deductions of circulating tumor cells^16^. The integer copy-number values were then used to calculate the Pearson correlation with gene expression and DNA methylation.

### CNV deduction with RNA-seq data

Approximately 6 000 genes, whose average relative expression level (calculated as log_2_ (FPKM + 1)) exceeded 1.5 across all single-cell samples, were used to measure CNVs according to a previous published method^11^. The CNV value for each gene was defined as the mean expression level (FPKM) of the 100 genes around the gene (50 upstream genes and 50 downstream genes). The CNV values were then centered to zero by subtracting the average CNV value for each cell^11^. Furthermore, the relative CNV value of a given 10-Mb window was calculated by averaging the values of all the genes within the window. We performed a bulk RNA-seq analysis of liver tissues near the HCC tissue and also obtained normal liver bulk RNA-seq data from NCBI as the normal reference (accession number: ERX011229). Single-cell data sets were then normalized to the normal reference, and hierarchical clustering was performed to discriminate between samples based on the severity of copy-number abnormality.

### Sensitivity and specificity of CNV deductions

We used the whole-genome sequencing data of bulk HepG2 cells as a standard reference for the bulk HepG2 RRBS data. We then calculated the sensitivity and specificity of CNV deductions at different resolution levels (from 0.1 to 10 Mb) for bulk RRBS data. We assessed the specificity and sensitivity of CNV deduction in scTrio-seq data using a normal diploid cell line (mouse mESCs). The normalized copy-number value of each window was expected to be within the range of (1.5-2.5) for autosomes in specificity calculation, and (0.5-1.5) for X chromosome in sensitivity calculation.

### DNA methylation variance among single cells

We estimated the cell-to-cell variance with a 3 000-bp window as described in a previously published study^20^. First, the mean methylation rate of each window in each cell was calculated, and the reciprocal of the SEM for each sample was set as the weight value for calculating the variance among different cells. The lower bound of the chi-squared confidence interval of the variance estimator with a confidence level of 0.95 was then used to calculate the variances in each genome element. The variable windows were then ranked with their variable values. Distribution enrichment of each genomic element in the top 300 variable windows were calculated and the significance was checked using Fisher's exact test.

### Identification of dmCGIs between two HCC subpopulations

For the following analysis, we selected definitively methylated CpG (mCG) sites or unmethylated (umCG) sites that were covered at least three times in a sample. We counted the mCG and umCG in each CGI and determined its methylation level by calculating the ratio between mCG and total CpGs. Only the CGIs that had at least five CpGs detected in a single-cell sample were considered as qualified. We then added the number of mCG and umCG sites in a CGI across samples in the same subpopulation if a CGI is qualified in 3 out of 7 cells in subpopulation I and in 5 out of 18 cells in subpopulation II. Sites that were differentially methylated at a significance level of 0.05 as determined by the Fisher's exact test and had a minimum methylation difference of 0.3 between two subpopulations were considered dmCGIs.

### Data access

All sequencing data have been submitted to the NCBI Gene Expression Omnibus (http://www.ncbi.nlm.nih.gov/geo/) under accession number GSE65364.

## Author Contributions

YuH, FT, YanH and JP designed the study, interpreted data and wrote the manuscript; YuH, HG, XL, XW and LW performed experiments and analyzed data; the bioinformatics analysis was performed by YuH, XL, CC, BH and PZ.

## Competing Financial Interests

The authors declare no conflict of interest.

## Figures and Tables

**Table 1:** Number of the detected CpG sites, genes, and MspI-digested fragments in single HepG2 cells

Sample	Unique CpGs (1×)	Unique CpGs (3×)	Genes (FPKM≥0.1)	Genes (FPKM≥1)	MspI-digested fragments
scTrio-HepG2-#1	1 834 536	1 276 842	6 083	4 373	150 288
scTrio-HepG2-#2	1 239 255	819 238	6 440	4 746	103 892
scTrio-HepG2-#3	1 217 007	709 874	6 271	5 122	104 884
scTrio-HepG2-#4	1 251 747	725 124	5 808	4 329	105 635
scTrio-HepG2-#5	1 762 799	1 201 953	6 437	4 904	145 361
scTrio-HepG2-#6	1 820 527	1 308 313	6 036	4 702	146 772
Mean of scTrio-HepG2	1 520 979	1 006 891	6 179	4 696	126 139
scRRBS-HepG2-#1	1 336 924	780 377	/	/	115 853
scRRBS-HepG2-#2	1 199 569	701 340	/	/	105 278
scRNA-HepG2-#1	/	/	6 099	4 335	/
scRNA-HepG2-#2	/	/	6 542	4 987	/

## Supplementary Material

Supplementary information, Table S1 Sequencing information of DNA methylome data.

Supplementary information, Table S2 Sequencing information of transcriptome data.

Supplementary information, Figure S1 Sensitivity and reliability of DNA methylome and transcriptome analysis in scTrio-seq data.

Supplementary information, Figure S2 CNV deduction using bulk RRBS data of HepG2 cells

Supplementary information, Figure S3 CNV deduction using scRRBS data and scTrio-seq data

Supplementary information, Figure S4 Correlations between DNA methylation, gene expression and DNA copy number in HepG2 cells.

Supplementary information, Figure S5 Correlations between DNA copy number and gene expression (or DNA methylation) in scTrio-seq data.

Supplementary information, Figure S6 DNA methylome differences between HCC bulk cells and liver bulk cells.

Supplementary information, Figure S7 DNA methylome of single HCC cells.

Supplementary information, Figure S8 Copy number variations of HCC cells.

Supplementary information, Figure S9 Differences between subpopulation I and subpopulation II HCC cells.

Supplementary information, Figure S10 DNA methylome heterogeneity among 25 HCC cells.

Supplementary information, Figure S11 Subpopulation I HCC cells lack complement and coagulation cascades pathway.
