# Supplementary material for: Single-cell triple omics sequencing reveals genetic, epigenetic, and transcriptomic heterogeneity in hepatocellular carcinomas
Source: Cell Res. 2016 Feb 23;26(3):304–19. doi: 10.1038/cr.2016.23 (PMC4783472; doi:10.1038/cr.2016.23)
Supplement: Supplementary information, Table S1 — Sequencing information of DNA methylome data. [file cr201623x1.pdf]

Supplementary Table S1. Sequencing information of DNA methylome data.

	Sample	Total number of reads analysed	Mapping efficiency	Total number of C's analysed	C--T conversion rate (lambda)	CpG_1X	CpG_3X	CpG (unmethylated or methylated) (Depth>=3)	Unmethylated or Methylated CpG rate (Depth>=3)	CpG_Mean methylation rate
**mES_scTrio-seq**	scTrio-mESC-#1	18,559,048	65.2%	300,404,591	0.997	895,586	695,037	649,344	0.93	0.32
	scTrio-mESC-#2	8,433,786	65.0%	135,227,440	0.994	489,971	361,190	346,709	0.96	0.35
	scTrio-mESC-#3	14,973,142	62.7%	234,908,660	0.995	1,351,353	1,113,202	1,043,322	0.94	0.30
	scTrio-mESC-#4	16,404,472	60.5%	245,969,158	0.993	843,936	660,617	621,523	0.94	0.29
	scTrio-mESC-#5	15,835,486	59.0%	233,045,244	0.994	562,586	402,585	380,800	0.95	0.29
	scTrio-mESC-#6	20,909,806	64.8%	338,412,495	0.995	704,162	533,219	493,179	0.92	0.34
	scRRBS-mESC-#1	14,878,276	62.7%	234,579,500	0.993	468,855	330,486	306,312	0.93	0.42
	scRRBS-mESC-#2	17,533,824	67.5%	297,882,198	0.994	562,864	411,751	374,251	0.91	0.32
**HepG2_scTrio-seq**	scTrio-HepG2-#1	20,789,093	65.6%	11,580,407	0.996	1,834,536	1,276,842	1,243,501	0.97	0.43
	scTrio-HepG2-#2	19,346,320	60.3%	7,711,443	0.995	1,239,255	819,238	802,355	0.98	0.41
	scTrio-HepG2-#3	23,631,568	61.2%	7,682,364	0.995	1,217,007	709,874	693,935	0.98	0.43
	scTrio-HepG2-#4	22,551,179	61.1%	7,802,920	0.994	1,251,747	725,124	705,724	0.97	0.43
	scTrio-HepG2-#5	24,771,046	62.4%	11,093,721	0.995	1,762,799	1,201,953	1,168,812	0.97	0.40
	scTrio-HepG2-#6	24,633,204	61.8%	11,393,492	0.993	1,820,527	1,308,313	1,273,075	0.97	0.44
	scRRBS-HepG2-#1	17,561,310	64.4%	8,548,125	0.995	1,336,924	780,377	764,503	0.98	0.44
	scRRBS-HepG2-#2	15,463,469	63.5%	7,659,425	0.994	1,199,569	701,340	687,994	0.98	0.44
**HCC_scTrio-seq**	HCC-sc#01	10,930,256	4.2%	11,429,840	0.991	472,832	210,793	206,462	0.98	0.27
	HCC-sc#02	13,172,102	11.3%	37,355,340	0.996	843,574	493,554	477,753	0.97	0.30
	HCC-sc#03	14,483,358	5.3%	19,169,268	0.992	628,343	317,681	310,006	0.98	0.33
	HCC-sc#04	5,992,350	9.6%	13,291,853	0.989	681,492	256,942	247,898	0.96	0.32
	HCC-sc#05	15,819,498	5.5%	21,677,294	0.994	527,455	298,165	292,059	0.98	0.28
	HCC-sc#06	11,630,242	3.3%	9,743,596	0.999	330,823	151,971	149,471	0.98	0.35
	HCC-sc#07	11,625,610	3.3%	9,712,543	0.994	283,503	147,472	145,180	0.98	0.32
	HCC-sc#08	12,067,398	2.5%	7,501,123	0.993	242,245	116,291	114,594	0.99	0.31
	HCC-sc#09	10,661,770	4.2%	11,254,743	0.994	510,188	204,188	199,274	0.98	0.28
	HCC-sc#10	11,029,622	4.5%	12,695,526	0.996	511,699	222,398	217,634	0.98	0.29
	HCC-sc#11	15,416,150	3.5%	13,643,374	0.995	247,374	143,056	141,220	0.99	0.32
	HCC-sc#12	15,687,786	5.7%	21,707,100	0.991	479,749	283,633	278,759	0.98	0.31
	HCC-sc#13	23,123,218	8.8%	25,031,373	0.979	728,875	455,594	449,005	0.99	0.38
	HCC-sc#14	19,777,660	2.6%	12,742,483	0.989	256,823	146,553	144,862	0.99	0.32
	HCC-sc#15	16,006,206	3.8%	14,133,978	0.994	328,936	182,407	179,873	0.99	0.37
	HCC-sc#16	19,124,898	10.7%	48,180,111	0.994	1,273,003	715,281	688,579	0.96	0.33
	HCC-sc#17	12,091,904	3.3%	10,125,381	0.997	141,461	80,148	79,240	0.99	0.35
	HCC-sc#18	14,456,256	5.9%	20,760,065	0.993	553,940	309,481	303,599	0.98	0.35
	HCC-sc#19	12,977,832	5.6%	17,989,162	0.982	450,254	253,157	247,953	0.98	0.34
	HCC-sc#20	13,212,662	5.8%	18,218,022	0.996	561,443	291,968	285,617	0.98	0.35
	HCC-sc#21	14,541,826	3.8%	13,200,496	0.998	361,179	182,410	179,580	0.98	0.37
	HCC-sc#22	14,132,888	5.6%	18,897,731	0.994	547,458	293,303	285,659	0.97	0.35
	HCC-sc#23	16,779,118	4.8%	19,842,507	0.997	450,245	262,511	257,248	0.98	0.32
	HCC-sc#24	17,818,916	2.9%	13,314,166	0.995	307,970	176,922	174,185	0.98	0.33
	HCC-sc#25	14,792,196	3.8%	12,827,391	0.997	476,988	219,458	214,988	0.98	0.33
	HCC-sc#26	16,083,912	3.2%	13,038,379	0.997	228,381	131,774	129,496	0.98	0.56
**HepG2_bulk_RRBS**	HepG2_bulk_RRBS	19,976,325	65.0%	71,774,902	0.993	9,544,039	7,520,487	\	\	0.43
**Liver_bulk_RRBS**	Liver_bulk_RRBS	17,008,946	71.0%	130,200,230	0.994	4,744,229	3,335,523	\	\	0.74
**HCC_bulk_RRBS**	HCC_bulk_RRBS	17,951,512	64.8%	183,144,058	0.995	5,116,243	3,478,045	\	\	0.62
