# Supplementary material for: Single-cell triple omics sequencing reveals genetic, epigenetic, and transcriptomic heterogeneity in hepatocellular carcinomas
Source: Cell Res. 2016 Feb 23;26(3):304–19. doi: 10.1038/cr.2016.23 (PMC4783472; doi:10.1038/cr.2016.23)
Supplement: Supplementary information, Table S2 — Sequencing information of transcriptome data. [file cr201623x2.pdf]

Supplementary Table S2. Sequencing information of transcriptome data.

	Sample	Total number of reads analysed	Mapped read pairs	Mapping efficiency	Mapped_genes_(FPKM>0)	Mapped_genes_(FPKM>=0.1)	Mapped_genes_(FPKM>=1)
**mES_scTrio-seq**	scTrio-mESC-#1	3,798,543	2,945,984	76.2%	8,603	8,383	7,567
	scTrio-mESC-#2	3,500,306	2,588,877	72.8%	5,572	5,259	4,592
	scTrio-mESC-#3	4,564,107	3,505,936	75.5%	7,665	7,269	6,428
	scTrio-mESC-#4	3,309,676	2,443,962	72.8%	5,569	5,191	4,269
	scTrio-mESC-#5	3,634,838	2,797,160	75.7%	7,638	7,379	6,573
	scTrio-mESC-#6	3,246,144	2,450,402	74.4%	5,900	5,699	5,126
	scRNA-mESC-#1	4,611,525	3,868,619	82.8%	9,840	9,635	8,649
	scRNA-mESC-#2	4,195,696	3,215,055	75.5%	9,576	9,359	8,344
**HepG2_scTrio-seq**	scTrio-HepG2-#1	3,045,084	1,462,211	46.0%	6,418	6,083	4,373
	scTrio-HepG2-#2	4,834,513	3,395,215	68.7%	7,378	6,440	4,746
	scTrio-HepG2-#3	7,674,252	5,887,868	76.0%	7,671	6,271	5,122
	scTrio-HepG2-#4	7,516,386	5,941,027	78.4%	7,548	5,808	4,329
	scTrio-HepG2-#5	5,256,450	3,347,958	61.9%	7,209	6,437	4,904
	scTrio-HepG2-#6	7,335,580	5,716,272	77.4%	7,528	6,036	4,702
	scRNA-HepG2-#1	4,709,407	3,487,995	73.1%	7,237	6,099	4,335
	scRNA-HepG2-#2	4,941,398	3,601,688	71.8%	7,536	6,542	4,987
**HCC_scTrio-seq**	HCC-sc#01	2,828,627	1,963,696	67.8%	5,848	5,640	4,933
	HCC-sc#02	3,083,187	2,193,106	70.0%	9,111	8,861	7,833
	HCC-sc#03	2,766,004	1,939,452	68.8%	7,304	7,084	6,163
	HCC-sc#04	2,758,456	1,944,950	69.0%	9,975	9,677	8,107
	HCC-sc#05	2,988,070	2,080,926	68.2%	8,397	8,167	7,151
	HCC-sc#06	4,712,912	3,420,401	70.5%	4,898	4,456	3,579
	HCC-sc#07	3,550,891	2,535,836	69.1%	3,161	2,874	2,214
	HCC-sc#08	2,410,810	1,632,619	66.7%	5,618	5,294	4,422
	HCC-sc#09	2,419,839	1,673,644	67.5%	6,386	6,130	5,369
	HCC-sc#10	2,543,524	1,758,820	67.7%	7,324	7,142	6,341
	HCC-sc#11	2,388,828	1,622,740	66.4%	4,985	4,786	4,082
	HCC-sc#12	4,722,051	3,809,203	79.2%	7,795	7,108	5,816
	HCC-sc#13	3,481,467	2,397,695	66.9%	5,182	4,636	3,222
	HCC-sc#14	4,373,642	3,292,304	74.0%	3,810	3,505	3,040
	HCC-sc#15	4,780,187	3,780,227	76.6%	6,733	6,201	4,951
	HCC-sc#16	8,509,635	7,023,513	81.1%	8,330	7,700	6,555
	HCC-sc#17	5,876,631	4,607,898	76.7%	5,200	4,724	4,169
	HCC-sc#18	4,519,007	3,402,922	73.6%	7,916	7,568	6,564
	HCC-sc#19	5,282,401	3,983,854	73.9%	6,921	6,604	5,809
	HCC-sc#20	4,283,905	3,061,573	69.3%	5,880	5,191	3,891
	HCC-sc#21	3,345,248	2,230,131	64.0%	2,758	2,497	1,831
	HCC-sc#22	4,888,827	3,365,506	67.2%	7,868	7,500	6,585
	HCC-sc#23	6,774,707	4,755,064	68.9%	8,231	7,764	6,837
	HCC-sc#24	1,330,117	907,944	66.7%	4,196	4,093	3,673
	HCC-sc#25	1,848,103	1,204,697	63.7%	4,286	4,160	3,430
	HCC-sc#26	4,395,881	2,562,311	57.8%	2,580	2,269	1,741
HepG2_bulk_RNA	HepG2_bulk_RNA	11,461,297	9,094,634	79.4%	15,559	14,046	11,332
HCC_bulk_RNA	HCC_bulk_RNA	9,209,167	8,094,858	87.9%	16,469	15,059	11,754
Liver_bulk_RNA	Liver_bulk_RNA	6,107,211	4,971,270	81.4%	15,644	14,405	9,198
