# Supplementary material for: Single-cell triple omics sequencing reveals genetic, epigenetic, and transcriptomic heterogeneity in hepatocellular carcinomas
Source: Cell Res. 2016 Feb 23;26(3):304–19. doi: 10.1038/cr.2016.23 (PMC4783472; doi:10.1038/cr.2016.23)
Supplement: Supplementary information, Figure S1 — Sensitivity and reliability of DNA methylome and transcriptome analysis in scTrio-seq data. [file cr201623x3.pdf]

Supplementary Figure 1

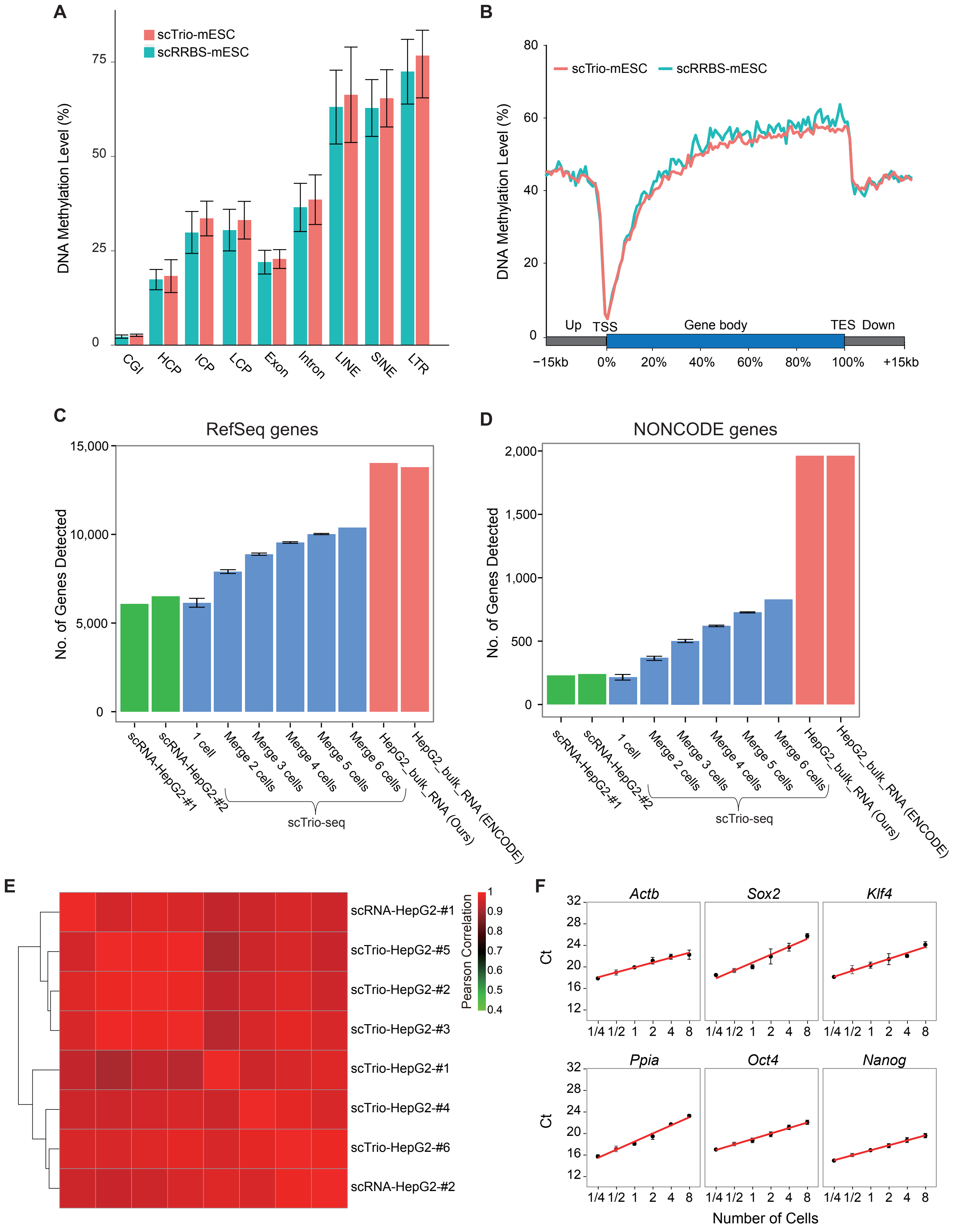

Supplementary information, Figure S1. Sensitivity and reliability of DNA methylome and transcriptome analysis in scTrio-seq data.

(A) The average DNA methylation levels of CpG sites in different genomic regions as determined from single mESCs methylome data.

(B) DNA methylation pattern in gene body regions as determined from single mESCs methylome data. The averaged methylation level of CpG sites was calculated from all RefSeq genes in regions from TSSs to TESs and their 15-kb flanking regions.

(C) Number of detected RefSeq genes in scRNA-seq data, scTrio-seq data and two bulk RNA-seq data of HepG2 cells.

(D) Number of detected NONCODE genes in scRNA-seq data, scTrio-seq data and two bulk RNA-seq data of HepG2 cells.

(E) Unsupervised hierarchical clustering analysis based on the Pearson correlations of gene expression between single mESC samples.

(F) Quantitative accuracy of RNA detection in scTrio-seq. Real-time quantitative PCR cycle threshold (Ct) results of housekeeping genes and pluripotent genes are shown. The dots show the mean values, and error bars represent the standard deviations of four biological replicates. The cell lysis products were serially diluted prior to amplification of cDNA.
