# Supplementary material for: Single-cell triple omics sequencing reveals genetic, epigenetic, and transcriptomic heterogeneity in hepatocellular carcinomas
Source: Cell Res. 2016 Feb 23;26(3):304–19. doi: 10.1038/cr.2016.23 (PMC4783472; doi:10.1038/cr.2016.23)
Supplement: Supplementary information, Figure S2 — CNV deduction using bulk RRBS data of HepG2 cells [file cr201623x4.pdf]

Supplementary Figure 2

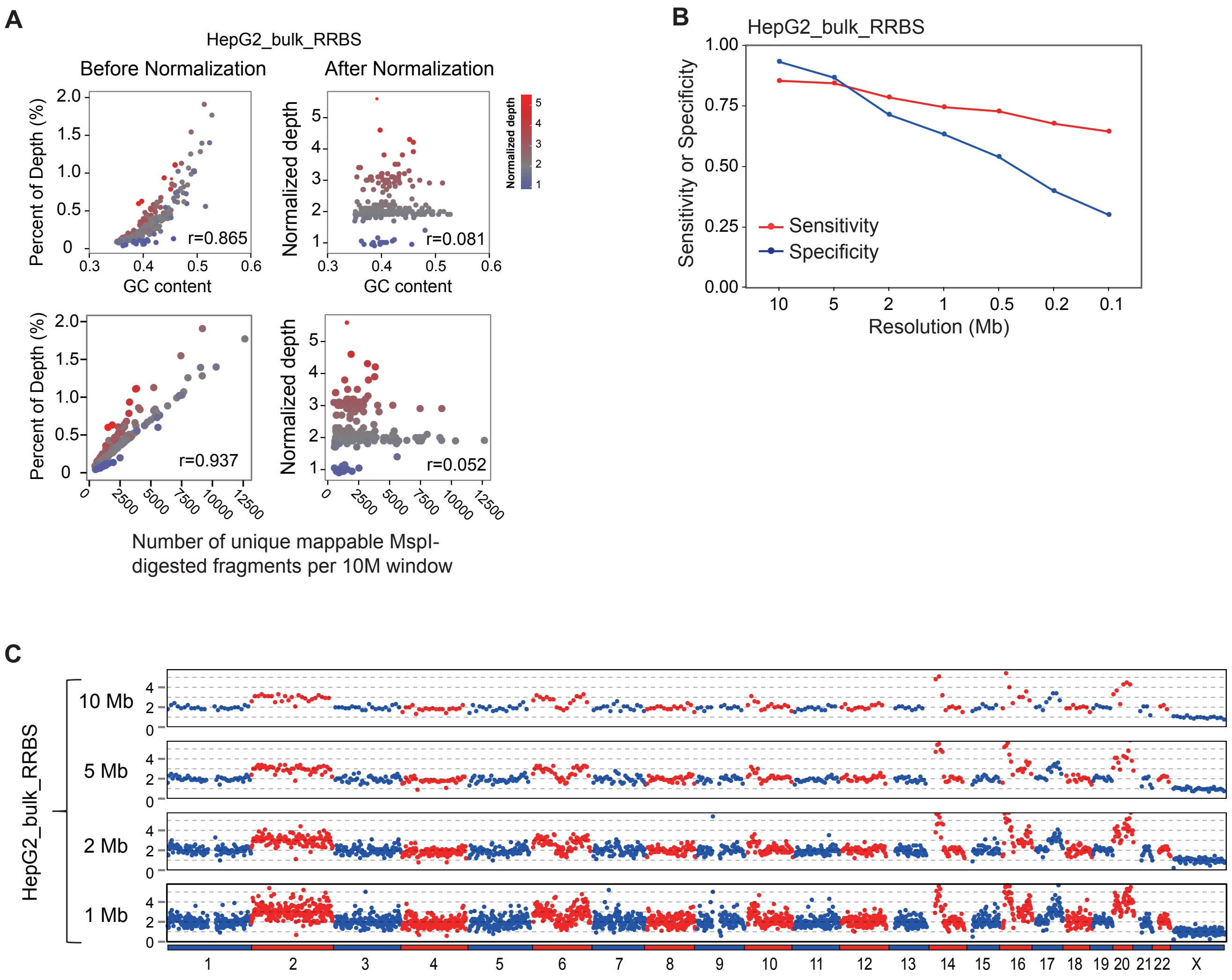

Supplementary information, Figure S2. CNV deduction using bulk RRBS data of HepG2 cells

(A) The strong biases of sequencing depth on GC content and MspI-digested fragments are eliminated after normalization. The resolution is 10-megabases when calculating the GC content and number of unique mappable MspI-digested fragments.

(B) Sensitivity and specificity of CNV deductions determined from HepG2 bulk RRBS data at different resolution levels.

(C) Copy number variation deduction results of HepG2 bulk RRBS data at different resolution levels.
