# Supplementary material for: Single-cell triple omics sequencing reveals genetic, epigenetic, and transcriptomic heterogeneity in hepatocellular carcinomas
Source: Cell Res. 2016 Feb 23;26(3):304–19. doi: 10.1038/cr.2016.23 (PMC4783472; doi:10.1038/cr.2016.23)
Supplement: Supplementary information, Figure S3 — CNV deduction using scRRBS data and scTrio-seq data [file cr201623x5.pdf]

Supplementary Figure 3

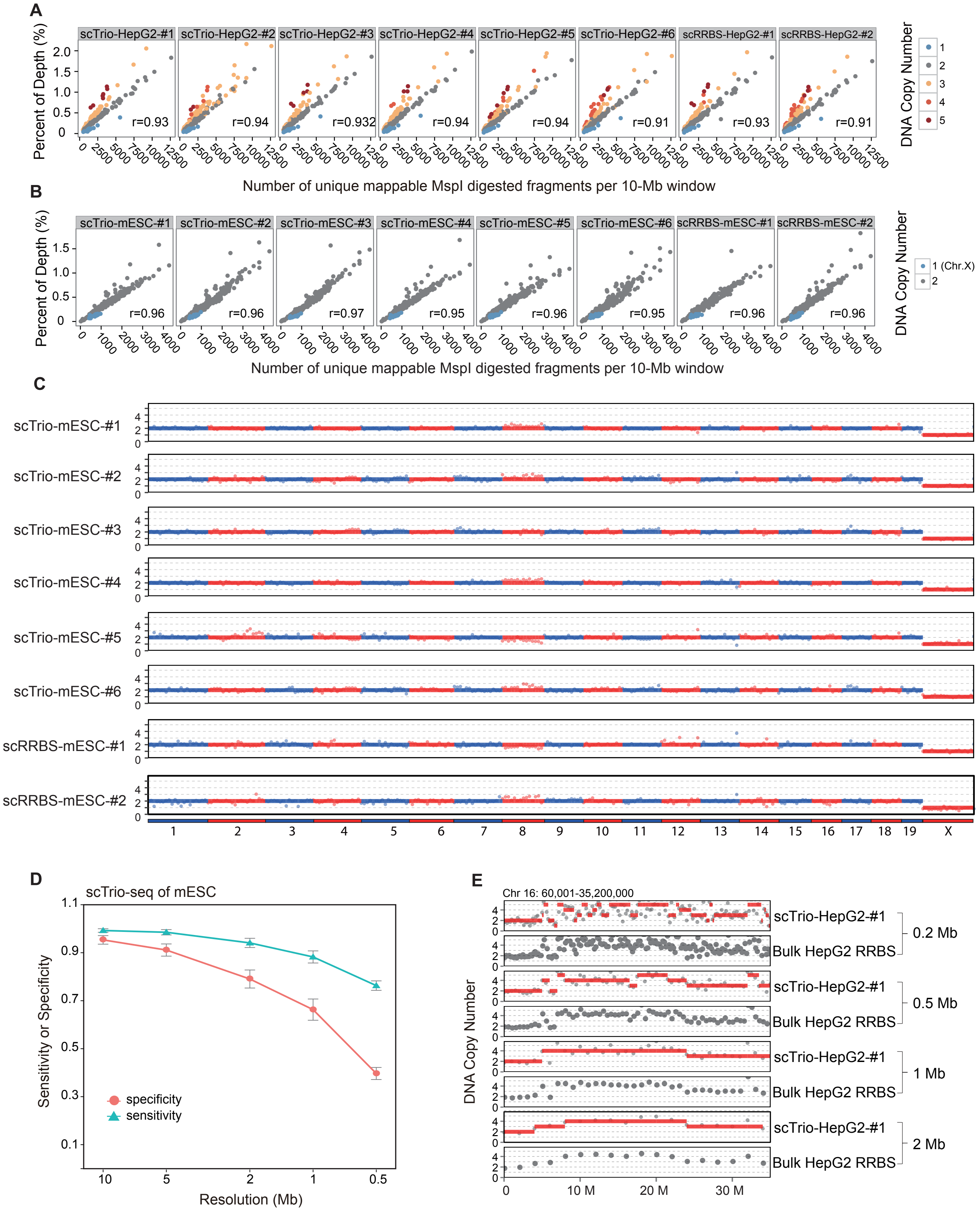

Supplementary information, Figure S3. CNV deduction using scRRBS data and scTrio-seq data

(A) The correlations between the sequencing depth and the number of unique mappable MspI-digested fragments of HepG2 scTrio-seq data or scRRBS data. The resolution is 10-megabases. The regions with the copy number of one include the chromosome X.

(B) The correlations between the sequencing depth and the number of unique mappable MspI-digested fragments of mESC scTrio-seq data or scRRBS data. The resolution is 10-megabases. The regions with the copy number of one are on chromosome X.

(C) Copy number variation deduction results of single mESC cells at a 10-Mb resolution. The red or blue dots represent the normalized copy number values and the red or blue segments show the HMM results.

(D) Specificity and sensitivity of CNV deductions determined from single mESC data at different resolution levels. Error bar represents the standard error of 8 mESC cells. The regions with copy number of one on chromosome X were regarded as true positive values when calculating the sensitivity.

(E) Zoom in pictures show shorter CNV segments on the p arm of Chr. 16 in HepG2 cells. The red segments represent the HMM results in scTrio-seq data. Bulk HepG2 RRBS data was used as a standard control.
