# Supplementary material for: Single-cell triple omics sequencing reveals genetic, epigenetic, and transcriptomic heterogeneity in hepatocellular carcinomas
Source: Cell Res. 2016 Feb 23;26(3):304–19. doi: 10.1038/cr.2016.23 (PMC4783472; doi:10.1038/cr.2016.23)
Supplement: Supplementary information, Figure S4 — Correlations between DNA methylation, gene expression and DNA copy number in HepG2 cells. [file cr201623x6.pdf]

Supplementary Figure 4

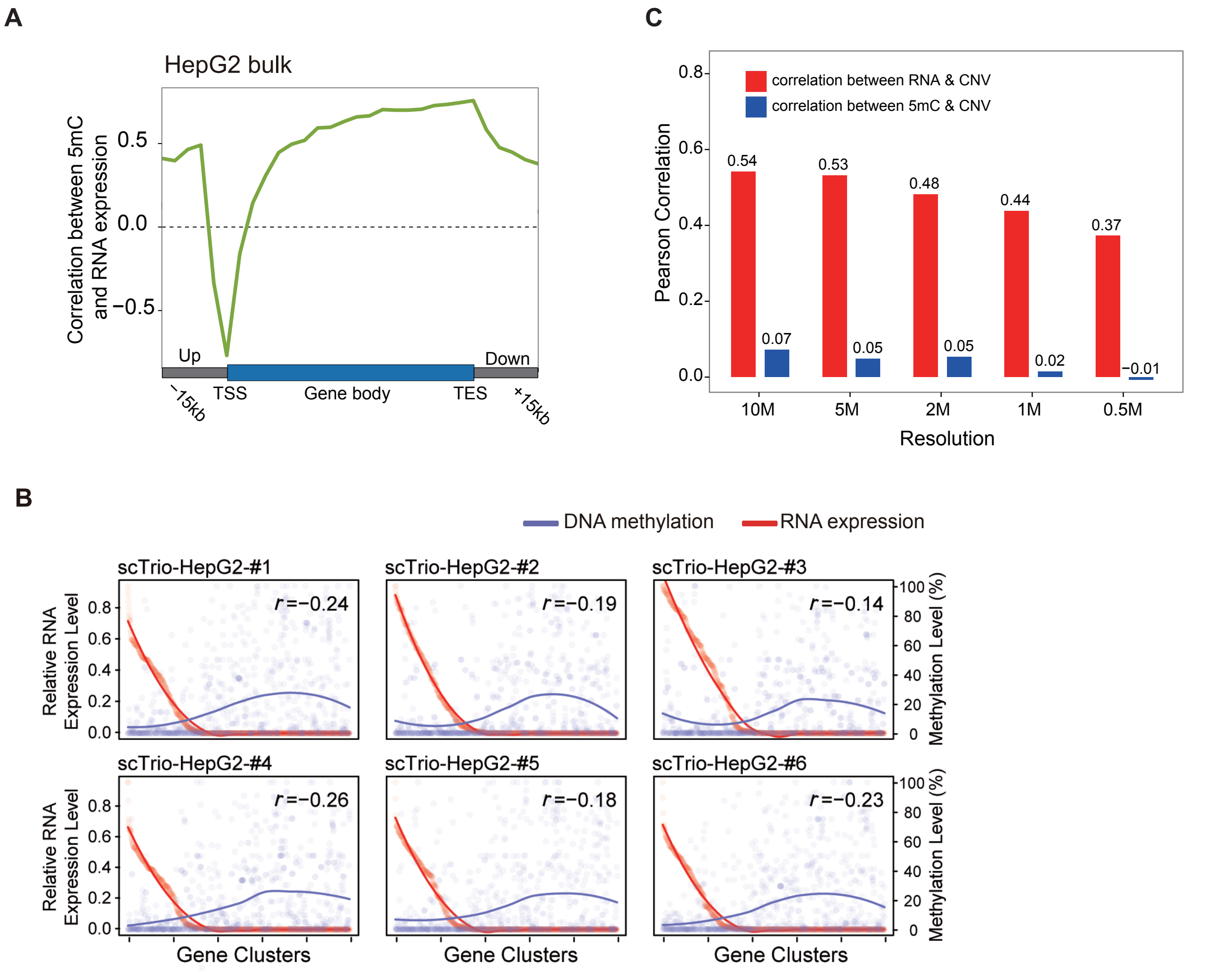

Supplementary information, Figure S4. Correlations between DNA methylation, gene expression and DNA copy number in HepG2 cells.

(A) The Pearson correlations between DNA methylation and gene expression were calculated on different regions on gene body (from TSS to TES) and their 15-kb flanking regions of HepG2 bulk cells data.

(B) Scatter plot of the relative gene expression levels (red dot and line) and DNA methylation levels (blue dot and line) in the corresponding genes' promoter regions in scTrio-seq data. The relative RNA expression levels are normalized $\log_2$ (FPKM +1), whose values are normalized to 0~1 by dividing with the max value. The genes were ranked by their relative expression levels in HepG2 bulk sample, and the genes' FPKM values were reset to 0.001 when they were less than 0.001. The Pearson correlation coefficients (r) were calculated between the DNA methylation levels of gene promoters and relative gene expression levels.

(C) The Pearson correlations between DNA copy number and gene expression (or DNA methylation) in bulk HepG2 cells at different resolutions.
