# Supplementary material for: Single-cell triple omics sequencing reveals genetic, epigenetic, and transcriptomic heterogeneity in hepatocellular carcinomas
Source: Cell Res. 2016 Feb 23;26(3):304–19. doi: 10.1038/cr.2016.23 (PMC4783472; doi:10.1038/cr.2016.23)
Supplement: Supplementary information, Figure S5 — Correlations between DNA copy number and gene expression (or DNA methylation) in scTrio-seq data. [file cr201623x7.pdf]

Supplementary Figure 5

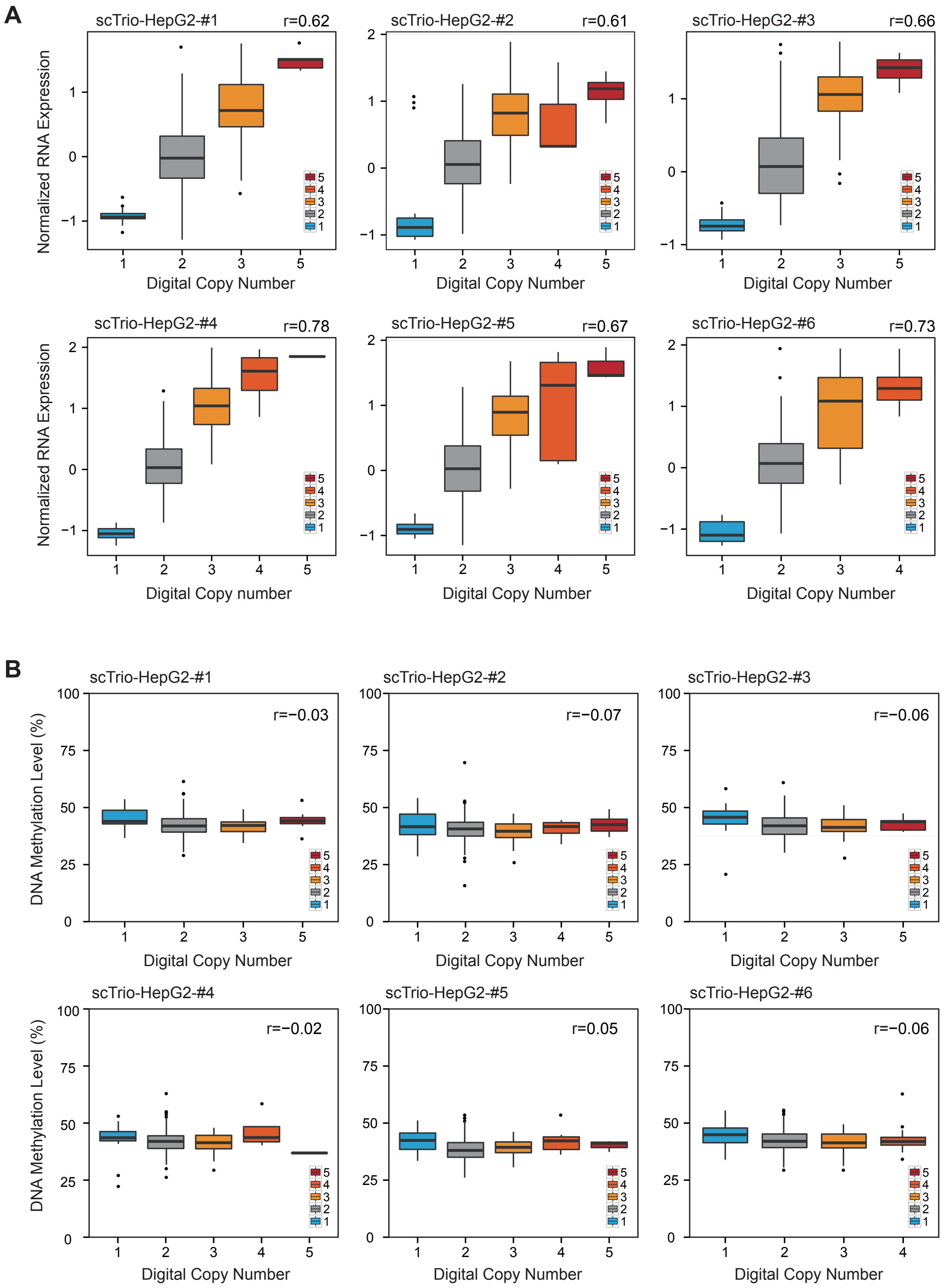

Supplementary information, Figure S5. Correlations between DNA copy number and gene expression (or DNA methylation) in scTrio-seq data.

(A) The correlations between the integer copy numbers and gene expressions at a 10-Mb resolution. The boxplot shows the distributions of 10-Mb windows' relative expression level within each copy number group. The Pearson correlation coefficient is shown at the top right corner.

(B) The correlations between the integer copy numbers and DNA methylation level at 10-Mb resolution. The boxplot show the distributions of 10-Mb windows' methylation level within each copy number group. The Pearson correlation was labeled on the top right corner.
