# Supplementary material for: Single-cell triple omics sequencing reveals genetic, epigenetic, and transcriptomic heterogeneity in hepatocellular carcinomas
Source: Cell Res. 2016 Feb 23;26(3):304–19. doi: 10.1038/cr.2016.23 (PMC4783472; doi:10.1038/cr.2016.23)
Supplement: Supplementary information, Figure S6 — DNA methylome differences between HCC bulk cells and liver bulk cells. [file cr201623x8.pdf]

Supplementary Figure 6

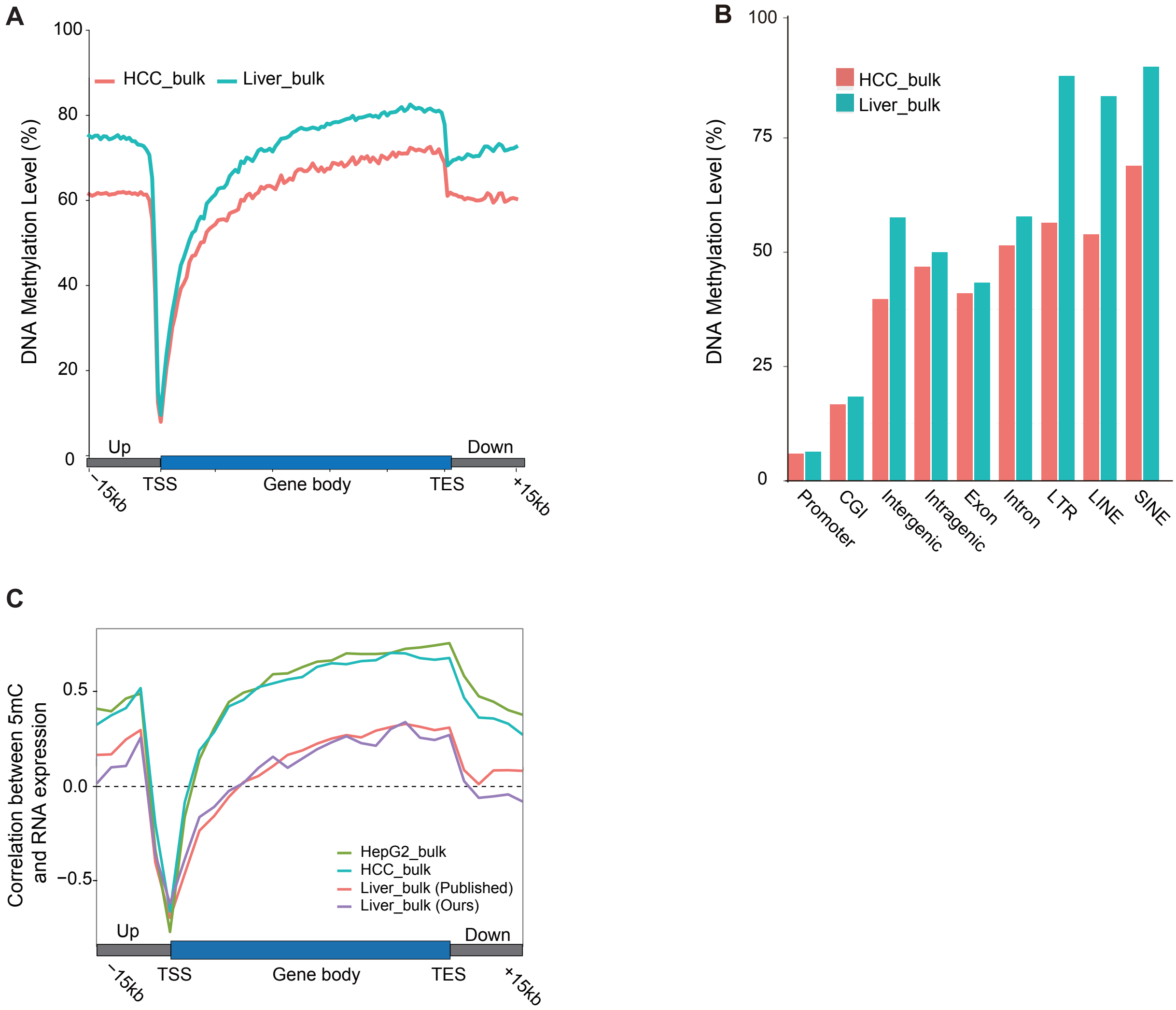

Supplementary information, Figure S6. DNA methylome differences between HCC bulk cells and liver bulk cells.

(A) The average DNA methylation levels from TSSs to TESs of all RefSeq genes and their 15-kb flanking regions in HCC and liver bulk RRBS data.

(B) The average DNA methylation levels in different genomic regions as determined from bulk HCC and liver RRBS data.

(C) The Pearson correlation between DNA methylation and gene expression was calculated on different regions in gene body (from TSS to TES) and their 15-kb flanking regions in bulk cell data. The correlations of adult liver cells were calculated using the published whole genome bisulfite sequencing data of adult liver cells (GSM916049) and the published RNA-seq data of human liver (accession number: ERX011229).
