# Supplementary material for: Single-cell triple omics sequencing reveals genetic, epigenetic, and transcriptomic heterogeneity in hepatocellular carcinomas
Source: Cell Res. 2016 Feb 23;26(3):304–19. doi: 10.1038/cr.2016.23 (PMC4783472; doi:10.1038/cr.2016.23)
Supplement: Supplementary information, Figure S7 — DNA methylome of single HCC cells. [file cr201623x9.pdf]

Supplementary Figure 7

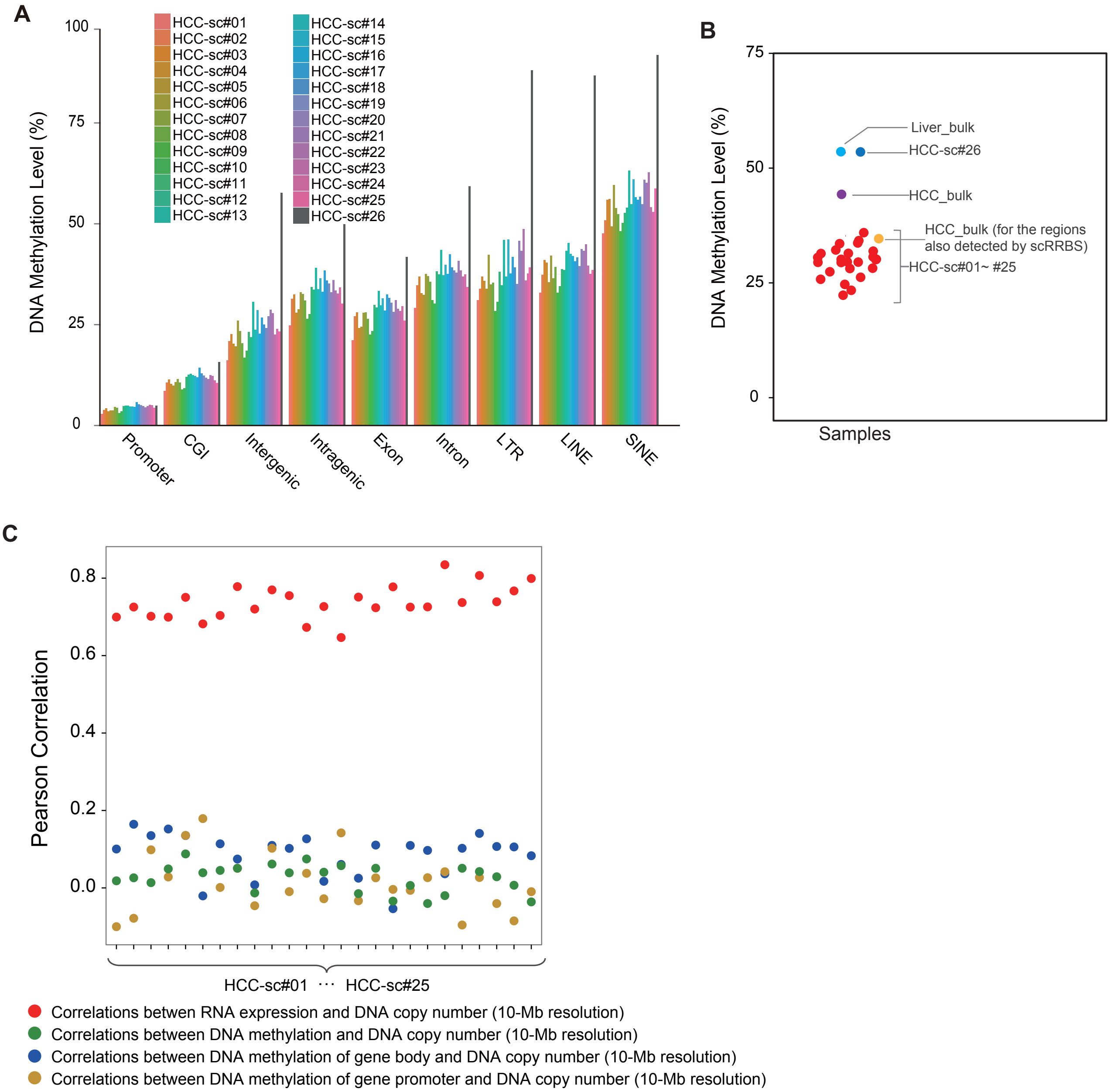


**Supplementary information, Figure S7. DNA methylome of single HCC cells.**

**(A)** The average DNA methylation levels of CpG sites in different genomic regions as determined from HCC scTrio-seq data. The HCC-sc#26 cell which shows much higher DNA methylation level was likely a normal cell in HCC tissue.

**(B)** The global methylation levels of all the single HCC cells as well as HCC bulk and liver bulk samples. The HCC_bulk (for the regions also detected by scRRBS) represents the DNA methylation of HCC_bulk cells, the calculation for which only includes regions that are also detected in HCC scTrio-seq data.

**(C)** Pearson correlation coefficient values between digital DNA copy number values and gene expression (and DNA methylation) in each HCC cells.
