# Supplementary material for: Single-cell triple omics sequencing reveals genetic, epigenetic, and transcriptomic heterogeneity in hepatocellular carcinomas
Source: Cell Res. 2016 Feb 23;26(3):304–19. doi: 10.1038/cr.2016.23 (PMC4783472; doi:10.1038/cr.2016.23)
Supplement: Supplementary information, Figure S8 — Copy number variations of HCC cells. [file cr201623x10.pdf]

Supplementary Figure 8

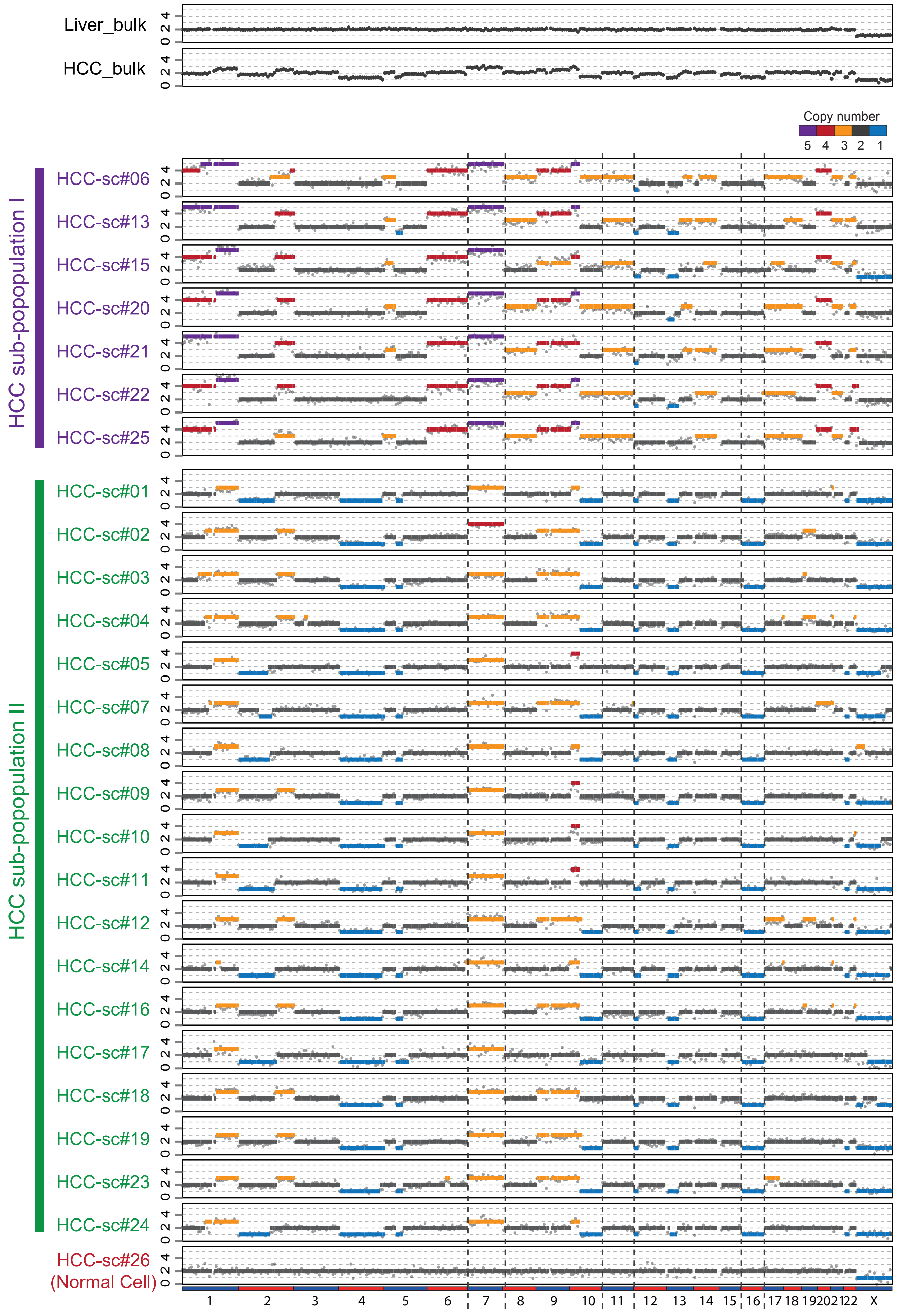

Supplementary information, Figure S8. Copy number variations of HCC cells.

Copy number variations deducted from bulk RRBS data or HCC scTrio-seq data. The resolution is 10 megabases. The grey dots represent the normalized copy numbers of each window, and the segments represent the integer copy number fitted by HMM.
