# Supplementary material for: Single-cell triple omics sequencing reveals genetic, epigenetic, and transcriptomic heterogeneity in hepatocellular carcinomas
Source: Cell Res. 2016 Feb 23;26(3):304–19. doi: 10.1038/cr.2016.23 (PMC4783472; doi:10.1038/cr.2016.23)
Supplement: Supplementary information, Figure S9 — Differences between subpopulation I and subpopulation II HCC cells. [file cr201623x11.pdf]

# Supplementary Figure 9

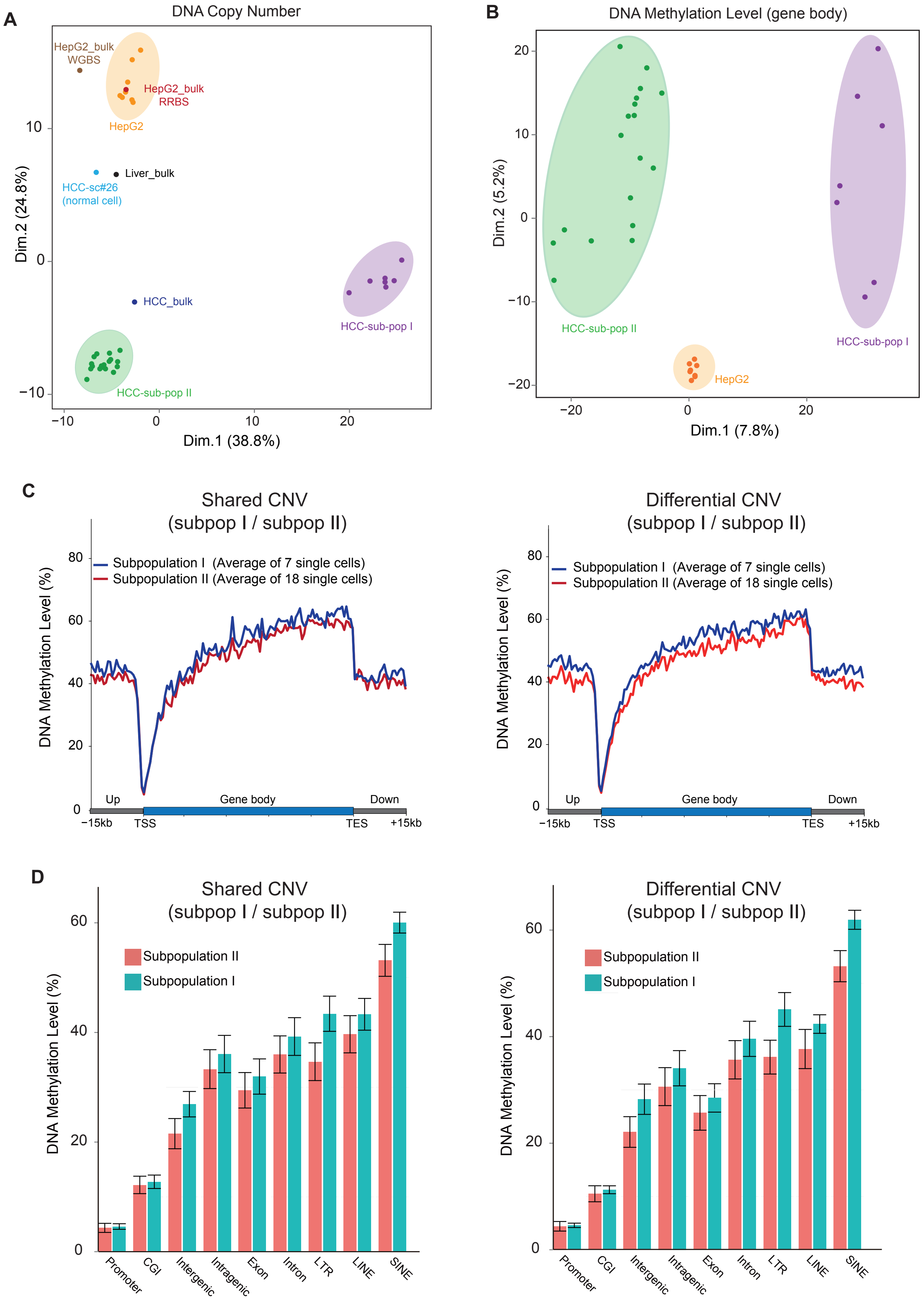

**Supplementary information, Figure S9. Differences between subpopulation I and subpopulation II HCC cells.**

**(A)** Principal component analysis of HepG2 cells and HCC cells according to the DNA copy number values of each 10-Mb window.

**(B)** Principal component analysis of HepG2 cells and HCC cells according to the gene body DNA methylation levels of each RefSeq gene.

**(C)** The average DNA methylation levels from TSSs to TESs of all RefSeq genes and their 15-kb flanking regions in subpopulation I and subpopulation II in HCC cells. Left, the genes that are located in the shared CNV regions; right, the genes that are located in the differential CNV regions.

**(D)** The average DNA methylation levels of different genomic regions in subpopulation I and subpopulation II in HCC cells. Left, the regions that are located in the shared CNV regions; right, the genes that are located in the differential CNV regions.
