# Supplementary material for: Single-cell triple omics sequencing reveals genetic, epigenetic, and transcriptomic heterogeneity in hepatocellular carcinomas
Source: Cell Res. 2016 Feb 23;26(3):304–19. doi: 10.1038/cr.2016.23 (PMC4783472; doi:10.1038/cr.2016.23)
Supplement: Supplementary information, Figure S10 — DNA methylome heterogeneity among 25 HCC cells. [file cr201623x12.pdf]

Supplementary Figure 10

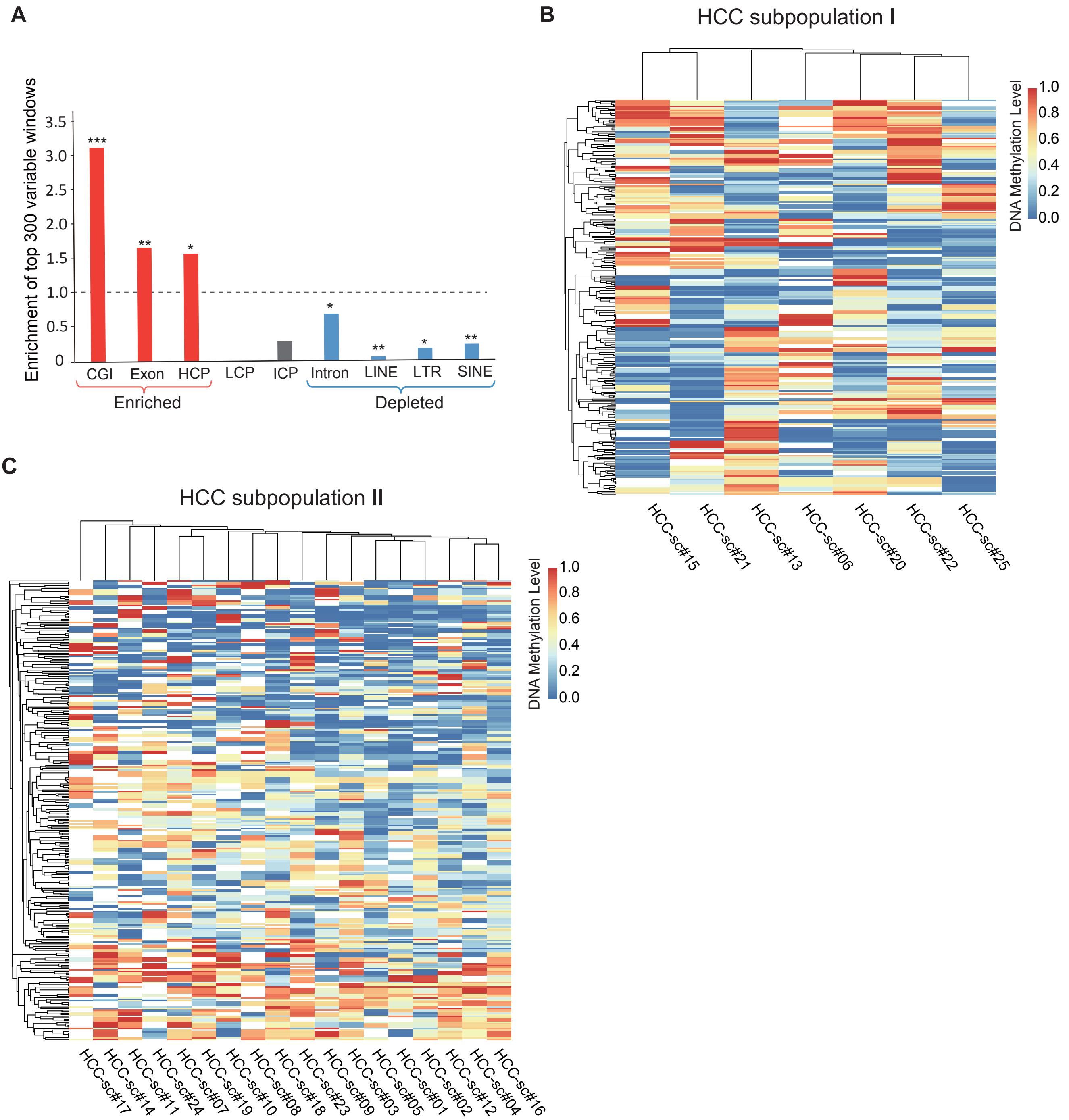

Supplementary information, Figure S10, DNA methylome heterogeneity among 25 HCC cells.

(A) Distribution enrichment of each genomic elements in the 300 most variable windows compared with all the variable windows. *: Fisher test FDR < 0.01; **: Fisher test FDR $<10^{-5}$; ***: Fisher test FDR $<10^{-10}$ (Fisher's exact test).

(B) The DNA methylation levels of top 300 variable windows in subpopulation I HCC cells. The genes and samples were arranged using Hierarchical clustering, separately.

(C) The DNA methylation levels of top 300 variable windows in subpopulation II HCC cells. The genes and samples were arranged using Hierarchical clustering, separately.
