# Supplementary material for: Single-cell triple omics sequencing reveals genetic, epigenetic, and transcriptomic heterogeneity in hepatocellular carcinomas
Source: Cell Res. 2016 Feb 23;26(3):304–19. doi: 10.1038/cr.2016.23 (PMC4783472; doi:10.1038/cr.2016.23)
Supplement: Supplementary information, Figure S11 — Subpopulation I HCC cells lack complement and coagulation cascades pathway. [file cr201623x13.pdf]

Supplementary Figure 11

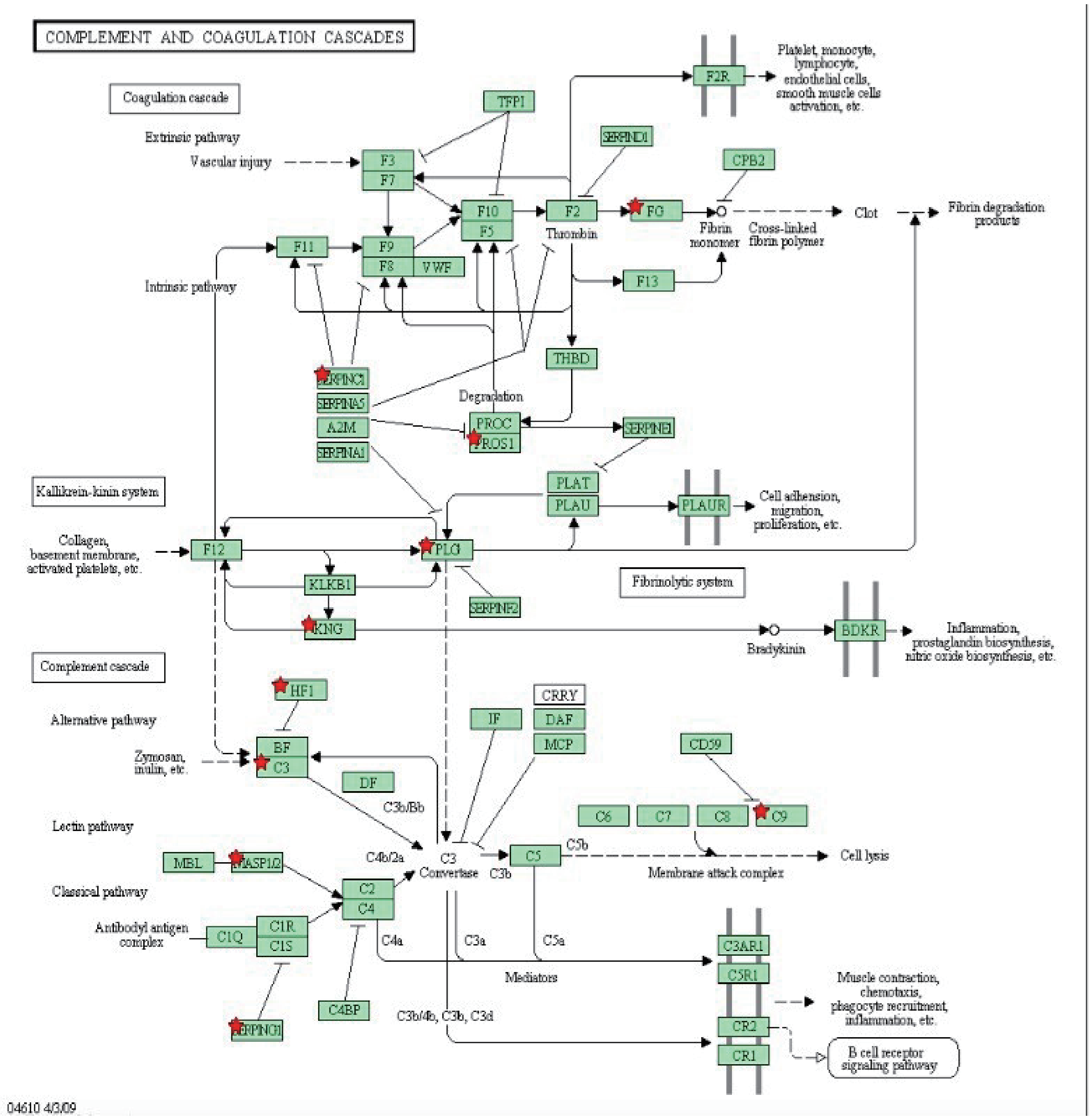

Supplementary information, Figure S11. Subpopulation I HCC cells lack complement and coagulation cascades pathway.

The complement and coagulation cascades from KEGG. The genes whose expressions are down regulated in subpopulation I HCC cells are marked with red starts.
